# Personalized adoptive immunotherapy for patients with EBV-associated tumors and complications: Evaluation of novel naturally processed and presented EBV-derived T-cell epitopes

**DOI:** 10.18632/oncotarget.23531

**Published:** 2017-12-21

**Authors:** Maren Bieling, Sabine Tischer, Ulrich Kalinke, Rainer Blasczyk, Søren Buus, Britta Maecker-Kolhoff, Britta Eiz-Vesper

**Affiliations:** ^1^ Institute for Transfusion Medicine, Hannover Medical School (MHH), Hanover, Germany; ^2^ Integrated Research and Treatment Center (IFB-Tx), MHH, Hanover, Germany; ^3^ Division of Experimental Infection Research, TWINCORE, Centre of Experimental and Clinical Infection Research, MHH, Hanover, Germany; ^4^ Department of Immunology and Microbiology, University of Copenhagen, Copenhagen, Denmark; ^5^ Department of Pediatric Hematology and Oncology, MHH, Hanover, Germany

**Keywords:** Epstein-Barr virus, post-transplant lymphoproliferative disease, adoptive T-cell immunotherapy, cytotoxic T-cell epitopes, T-cell monitoring

## Abstract

Morbidity and mortality of immunocompromised patients are increased by primary infection with or reactivation of Epstein-Barr virus (EBV), possibly triggering EBV^+^ post-transplant lymphoproliferative disease (PTLD). Adoptive transfer of EBV-specific cytotoxic T cells (EBV-CTLs) promises a non-toxic immunotherapy to effectively prevent or treat these complications.

To improve immunotherapy and immunomonitoring this study aimed at identifying and evaluating naturally processed and presented HLA-A*03:01-restricted EBV-CTL epitopes as immunodominant targets. More than 15000 peptides were sequenced from EBV-immortalized B cells transduced with soluble HLA-A*03:01, sorted using different epitope prediction tools and eleven candidates were preselected. T2 and Flex-T peptide-binding and dissociation assays confirmed the stability of peptide-MHC complexes. Their immunogenicity and clinical relevance were evaluated by assessing the frequencies and functionality of EBV-CTLs in healthy donors (*n* > 10) and EBV^+^ PTLD-patients (*n* = 5) by multimer staining, Eli- and FluoroSpot assays. All eleven peptides elicited EBV-CTL responses in the donors. Their clinical applicability was determined by small-scale T-cell enrichment using Cytokine Secretion Assay and immunophenotyping. Mixtures of these peptides when added to the EBV Consensus pool revealed enhanced stimulation and enrichment efficacy. These EBV-specific epitopes broadening the repertoire of known targets will improve manufacturing of clinically applicable EBV-CTLs and monitoring of EBV-specific T-cell responses in patients.

## INTRODUCTION

After hematopoietic stem cell (HSCT) and solid organ transplantation (SOT), patients are rendered highly susceptible to viral infections and reactivations due to transplant-related immunosuppressive therapy and delayed immune reconstitution. Therefore, infectious complications caused by endogenous herpes viruses like human cytomegalovirus (HCMV) and Epstein-Barr virus (EBV) or by lytic pathogens like human adenoviruses (hAdV) are common and lead to increasing morbidity and mortality in immunocompromised patients [1].

Worldwide, more than 90% of adults have been infected with EBV [2], ensuring latent persistence via immune evasion. In healthy humans, viral replication is controlled by effective cytotoxic T lymphocytes (CTLs) against viral gene products expressed during the latent or lytic reactivation cycles of EBV and against cellular antigens [3]. In immunocompromised patients, however, viral replication cannot be controlled due to insufficient EBV-specific immunity. Consequently, reactivations and/or outgrowth of latently EBV-infected B cells may occur leading to the development of high-grade lymphomas. Post-transplant lymphoproliferative disease (PTLD) represents the most common EBV-associated malignancy after transplantation. Up to 33% of primary infections post transplantation develop into true PTLD [4]. Reactivation of EBV in HSCT recipients ranges from 10 to 50%, while the incidences in SOT recipients vary from 1 to 20% depending on risk factors like EBV-serostatus, age at transplantation, immunosuppressive regimen post-transplant and organ graft [3, 5, 6]. The highest incidences following SOT (≤20%) have been detected in recipients of lung, small bowel or multiple organ grafts [3, 7, 8]. In general, EBV-seronegative patients with EBV-seropositive donors [7, 9] and EBV-seronegative transplant recipients suffering from primary EBV-infection in a post-transplant immunosuppressed setting [10, 11] carry the highest risk for PTLD development. Since 90% of pediatric and up to 70% of adult PTLDs [3, 12] are EBV-associated, the two main immunotherapeutic approaches currently aim at eliminating the EBV-infected target cells and at restoring the patients’ long-term EBV-specific immunity [3, 5, 13–18]. Despite success in several patients, treatment with the chimeric monoclonal anti-CD20 antibody, rituximab, as first line therapy, is related to an increased risk of further infections in B-cell-depleted patients because of hypogammaglobulinemia. In addition, it is likely to remain ineffective due to several host- and tumor-related mechanisms [5, 19–22] and frequently leads to PTLD recurrences [5, 23].

Adoptive T-cell immunotherapy with donor derived EBV-specific T cells focuses on administering EBV-specific T cells, which are specific to immunodominant latency-associated antigens expressed in PTLD, such as Epstein-Barr nuclear antigen 1 (EBNA1). In several clinical trials the adoptive transfer of functional EBV-specific T cells from completely or partially HLA-matched, EBV-seropositive donors has proven an effective, non-toxic immunotherapeutic approach to prevent and treat EBV-associated complications without increasing the risk of graft versus host disease (GvHD) [5, 13, 15–17, 22, 24].

The magnitude of T-cell responses to known immunodominant EBV-derived antigens (peptides, proteins and overlapping peptide pools), covering a wide range of HLA-restriction elements, considerably varies among EBV-seropositive healthy T-cell donors, thus indicating a high antigenic diversity [25–27]. Preliminary studies showed that 27% of EBV-seropositive healthy donors did not manifest immune responses to well-established EBV target antigens (e.g. immediate-early protein BZLF1 (BZLF1), latent membrane protein 2A (LMP2A) and EBNA1) [27]. Evaluated EBV-specific T-cell epitopes are thus less immunodominant than those of the phosphoprotein 65 (pp65) of HCMV and therefore not able to elicit an immune response in every single EBV-seropositive donor [25, 27]. Consequently, more than one EBV antigen is required for accurate monitoring of cellular immunity in patients, screening of potential T-cell donors and generation of effective EBV-specific T cells in sufficient numbers. This emphasizes the necessity of enlarging the repertoire of EBV-specific T-cell epitopes to both refine immunomonitoring and enhance multi-epitope-based EBV-specific immunotherapeutic strategies.

This study aims at the identification of novel HLA-A*03:01-restricted EBV-specific CD8^+^ T-cell epitopes presented *in vivo* by EBV-infected target cells. To ensure *in vivo* and clinical relevance, EBV-derived peptides were deliberately isolated from EBV-immortalized, HLA-A*03:01-lentivirally transduced B-lymphoblastoid cell lines (B-LCLs), acting as surrogate cells for PTLD [5]. Immunogenicity, cytotoxicity and clinical eligibility of eleven CTL candidate epitopes were evaluated. The newly identified, immunodominant EBV-specific CTL epitopes will improve (1) the accurate monitoring of EBV-specific T-cell immune responses in patients before and after transplantation, (2) the identification of suitable T-cell donors as well as (3) the manufacturing of clinical-grade antiviral T cells in a sufficient cell number for the adoptive transfer to ameliorate the clinical outcome of patients suffering from EBV-related complications.

**Table 1:** *In vivo* isolated, highly scored EBV-specific candidate-epitopes–*in silico* predicted results and IFN-γ EliSpot-based screening for immunogenicity

Sequence	Origin	Abbreviation	Epstein-Barr-Virus-Protein	Peptide-Ion-Score	NetMHC 4.0		NetCTL 1.2		NetMHCstab 1.0		ExPASy-ProtParam tool		SYF-PEITHI	Responders
[aa]	[B-LCL]		[UniProtKB-Database]	[pep_score]	[%Rank]	[BL]	[score]	[E]	[score]	[BL]	[Instab.- Index]	[class.]	[score]	[IFN-γ EliSpot (day 7)]
RLRAEAQVK		A*03_EBNA3A_RLRA_	Ebstein-Barr-Nuclear-Antigen-3A–EBNA-3A		0.40	SB	1.4208	E	0.633	SB HS	18.71	stable	36	9/18
KLLRYASAK	*in vivo* [024]	A*03_BPLF1_KLLR_	Large tegument protein deneddylase–BPLF1	13.57	0.01	SB	1.6755	E	0.785	SB HS	38.79	stable	35	5/14
TVARHLLGAK	*in vivo* [623]	A*03_BALF5_TVAR_	DNA polymerase catalytic protein - BALF5	13.30	0.15	SB	0.7951	E	0.586	SB WS	19.77	stable	26	7/14
ATGMVPAVKK	*in vivo* [623]	A*03_BBRF1_ATGM_	Portal protein UL6 homolog–BBRF1	28.73	0.20	SB	0.9726	E	0.431	WB	36.15	stable	20	2/10
KLVCSEPLVK	*in vivo* [024, 623]	A*03_BcRF1_KLVC_	TBP-like protein - BcRF1	30.29	0.40	SB	0.9152	E	0.597	WB WS	36.15	stable	31	5/14
**VTLAHAGYY**	***in vivo*** [1335]	A*03_BILF2_VTLA_ ^(1),(2)^	**Glycoprotein–BILF2**	**49.38**	**0.70**	**WB**	**1.2361**	**E**	**0.419**	**WB**	**–5.70**	**stable**	**14**	**13/21**
**FLLAMTSLR**	***in vivo*** [623]	A*03_BcRF1_FLLA_ ^(1),(2)^	**TBP-like protein–BcRF1**	**12.90**	**0.70**	**WB**	**1.4480**	**E**	**0.347**	**WB**	**27.09**	**stable**	**21**	**13/19**
FLGKYIKVKK	*in vivo* [024]	A*03_BTRF1_FLGK_	*„uncharacterized protein“*–BTRF1	16.21	1.00	WB	1.1954	E	0.357	WB	–19.35	stable	24	5/10
**QVATEGLAK**	***in vivo*** [024]	A*03_BALF3_QVAT_ ^(1),(2)^	**Tripartite terminase subunit UL28 homolog–BALF3**	**18.17**	**1.20**	**WB**	**0.9267**	**E**	**0.414**	**WB WS**	**21.91**	**stable**	**30**	**12/19**
TLVDVRAIK	*in vivo* [623]	A*03_BaRF1_TLVD_	Ribonucleoside-diphosphate reductase small chain–BaRF1	16.60	1.20	WB	1.0387	E	0.415	WB	–17.24	stable	26	5/14
KIVTNILIY	*in vivo* [024]	A*03_gB_KIVT_	envelope glycoprotein B–gB	10.09	1.30	WB	1.2615	E	0.346	WB	34.11	stable	20	2/10
**LIIPNVTLAH**	***in vivo*** [1335]	A*03_BILF2_LIIP_^(2)^	**Glycoprotein–BILF2**	**49.38**	**4.00**		**0.7476**		**0.239**		**–10.86**	**stable**	**22**	**11/20**

[aa] = amino acid, [B-LCL] = B-lymphoblastoid cell line, ^(1)^ = component of EBV_Consensus+3P_MIX_, ^(2)^ = component of EBV_Consensus+4P_MIX_, [Ref.] = References, [pep_score] = peptide score (sequences’ probability of an existent match to a database entry), [BL] = Binding Level, [SB] = strong binder, [WB] = weak binder, [HS] = highly stable binder, [WS] = weakly stable binder, [score] = combined prediction score, [E] = identified as potential CTL epitope, [Instab.-Index] = Instability Index, [class.] = classification.

Overview of the eleven investigated HLA-A*03:01-restricted candidate-epitopes and the known A*03_EBNA3A_RLRA_ as reference epitope regarding their peptide sequences, origin, protein source, analyzed prediction scores (NetMHC 4.0, NetCTL 1.2, NetMHCstab 1.0, ExPASy-ProtParam tool, SYFPEITHI) and *in vitro* T-cell responses (IFN-γ EliSpot assay). All isolated sequences with a peptide-ion-score >10 [pep_score] that reached the highest scores in the subsequent predictions regarding their peptide-binding affinity and peptide-MHC complex stability as well as with a clinically relevant role in EBV-latency, -reactivation and/or potentially malignant transformation are included. Candidate-epitopes were classified as immunodominant (immunoresponse in ≥20% of donors) and highly immunodominant (immunoresponse in >50% of the donors, as typed in bold) assessed by *in vitro* IFN-γ EliSpot assay.

## RESULTS

### Verification of *in vivo* isolated HLA-A*03:01-restricted EBV-derived peptides

A combination of different epitope prediction tools was applied to scan the unfiltered sequences of HLA-A*03:01-restricted EBV-derived peptides isolated *in vivo*. The secretion of soluble HLA-A*03:01 (sHLA-A*03:01) in the transduced EBV^+^B-LCLs’ supernatants reached levels up to 6.85 μg/ml (mean: 4.15 ± 1.93 μg/ml). A total of about 15,000 unfiltered EBV-specific peptide-sequences were isolated *in vivo* (Supplementary Figure 1). Among these, only 4.49% of the sequences (*n* = 673) remained after the first sorting exclusively based on the peptide-ion-score. As this particular score is not completely congruous with the quality of the sequence's MS/MS-spectrum, this relatively low cut-off value was chosen [38]. Resulting from the cut-off value of 15%RANK (NetMHC) 32.4% (*n* = 218) of the 673 ranked sequences remained candidates. Subsequent to the scanning of the candidates by NetMHC, NetCTL and NetMHCstab, the 20 highest scoring sequences of each EBV^+^B-LCL or those classified as strong [SB] or weak binders [WB] (*n* = 63) were comparatively analyzed by ExPASy-ProtParam-tool and SYFPEITHI. 17.5% of the remaining sequences (*n* = 11) answered the additional criterion of not presenting any homologies to the human genome (Table 1). Most of them derive from proteins associated with either latency and/or reactivation or with potential to promote malignant transformation. In this context A*03_BTRF1_FLGK_ represents the only exception as it derives from EBV protein BTRF1 that has not been characterized yet. Considering the HLA-A*03:01 peptide supermotif with focus on the primary anchor positions P2 and P9 [45, 46], all eleven EBV-peptide sequences carry one of the highly preferred amino acids at P2 (A, I, L, T, V, M, S). Eight of them contain the typically preferred residues at P9 (K, R). Taking all the mentioned criteria into account, these eleven EBV-specific peptide-sequences continued to be potentially relevant as novel T-cell epitopes and therefore appropriate for further investigation (Table 1). Four of them were predicted as strong and six of them as weak binders (NetMHC). These predicted binding affinities were confirmed by SYFPEITHI-scores ranging from 20 to 31, except for A*03_BILF2_VTLA_. Ten EBV-derived sequences were predicted to be ‘potential CTL epitopes’ by NetCTL with combined scores ranging from 0.748 to 1.676. Stability of the pMHC complexes was considered to be either highly or weakly stable (NetMHCstab) in ten of the sequences, confirmed by the instability indices obtained from the ExPASy-ProtParam-tool, classifying all eleven sequences to be stable. In summary, eleven *in vivo* isolated HLA-A*03:01-restricted EBV-derived peptides (Table 1) were found to be potentially relevant according to their respective epitope prediction scores and were therefore further on investigated.

### Immunogenic potential and functionality of the eleven EBV-peptide-specific T-cell epitopes

#### Proof of immunodominance by IFN-γ secretion

**Figure 1:**
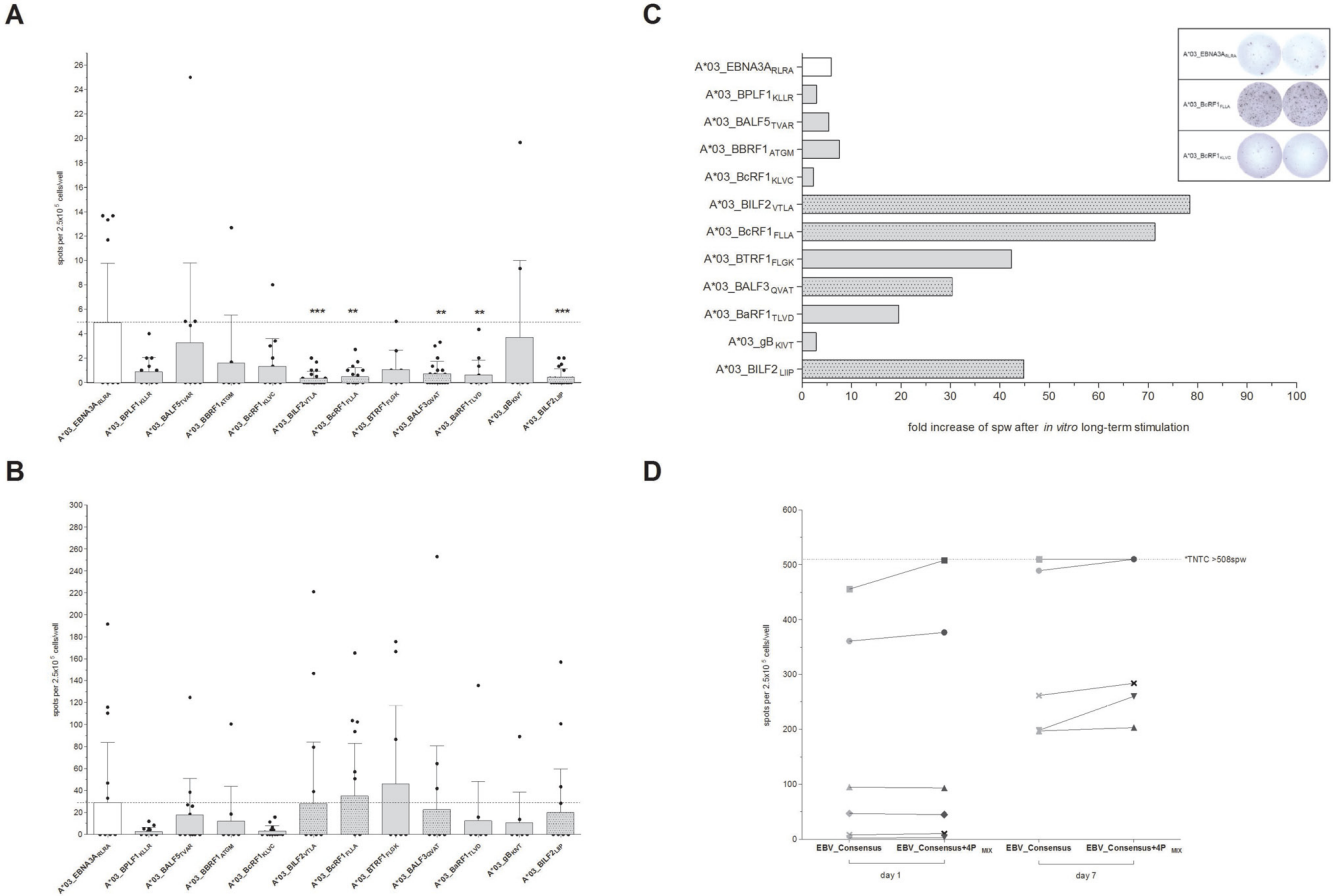
(**A**–**D**) Screening for HLA-A*03:01-restricted EBV-peptide-specific T cells by IFN-γ EliSpot. EBV-specific T-cell responses elicited by EBV-derived candidate-peptides were determined by IFN-γ EliSpot assays using PBMCs from healthy HLA-A*03:01-positive and EBV-seropositive donors (*n* ≥ 10). The known immunodominant HLA-A*03:01-specific T-cell epitope A*03_EBNA3A_RLRA_ and the available PepTivator EBV Consensus (EBV_Consensus) served as references. Results are indicated as the number of spots per well (spw) after the number of spw of the respective negative control has been subtracted from that one of the antigen well. Based on the cut-off value of >3 peptide-induced spw, donors were identified as positive responders. EBV-peptides initiating an immune response in >50% of the donors after seven days of *in vitro* stimulation were classified as highly immunodominant. IFN-γ EliSpot assays after (A) short-term (day 1) and (B) long-term (day 7) *in vitro* stimulation with one of the peptides. (C) The capacity of the EBV-peptides to generate EBV-specific T cells subsequent to long-term *in vitro* stimulation is separately demonstrated for each of the peptides by the resultant fold increases of spw. (D) The immunogenic potential of the respective peptides to reinforce available peptide pools (e.g. EBV_Consensus) was evaluated by means of comparing the resultant spw ensuing stimulation (‘day 1’ and ‘day 7’) with EBV_Consensus+4P_MIX_ (Table 1) to the respective values of the mere EBV_Consensus. Numbers of spw, too high to be individually detected by the EliSpot reader (>508 spw based on the highest determined count of spw), are indicated by the broken line. Results are displayed as individual results and means ± standard deviation (SD). Asterisks indicate statistically significant differences between the EBV-specific peptides and A*03_EBNA3A_RLRA_ (^**^p < 0.01, ^***^p < 0.001).

Peptides from the eleven, highest scoring sequences (Table 1) were then synthesized and screened in interferon-gamma (IFN-γ) EliSpot assay to evaluate their immunogenicity in short- (1 day) and long-term (7 days) *in vitro* stimulation assays (Figure 1). Unspecific T-cell responses to the evaluated peptides were not observed in peripheral blood mononuclear cells (PBMCs) from healthy donor controls (HLA-A*03:01^+^EBV^−^ or HLA-A*03:01^−^EBV^+^, data not shown). After short-term overnight *in vitro* stimulation only low numbers of spots per well (spw) could be detected ranging from 0.36 ± 0.59 (A*03_BILF2_VTLA_, *n* = 21) to 3.73 ± 6.27 spw (A*03_gB_KIVT_, *n* = 10, Figure 1A). In comparison to the known immunodominant reference peptide A*03_EBNA3A_RLRA_, the latter revealed a higher stimulating efficacy following short-term *in vitro* stimulation (4.93 ± 4.85 spw, *n* = 18, Figure 1A) than the newly identified peptides.

All eleven newly identified EBV-specific peptides were capable of inducing an EBV-specific T-cell response in ≥20% of the donors after 7 days of *in vitro* stimulation and were therefore classified as immunodominant (Table 1, Figure 1B). The long-term *in vitro* stimulation with A*03_EBNA3A_RLRA_ led to positive responses in 50% of the donors (*n* = 9/18) with specific T-cell responses ranging from 0.0 to 191.7 spw (29.1 **±** 54.5 spw, Figure 1B), representing an average increase of spw by 5.9-fold in comparison to day 1 (Figure 1C). IFN-γ EliSpot assays following long-term stimulation showed increased numbers of spw for the new epitopes, ranging from 2.67 **±** 3.82 (A*03_BPLF1_KLLR_) to 46.0 **±** 71.0 spw (A*03_BTRF1_FLGK_, Figure 1B) with an average increase of spw of 28-fold (Figure 1C). The peptides A*03_BILF2_VTLA_, A*03_BcRF1_FLLA_, A*03_BALF3_QVAT_ and A*03_BILF2_LIIP_ elicited a specific T-cell response in more than 50% of the assessed donors and were consequently classified as highly immunodominant with specific response rates of up to 68.4% (A*03_BcRF1_FLLA_, *n* = 19, Table 1). The highest number of spw of these epitopes was determined following the EBV-peptide-specific stimulation with A*03_BcRF1_FLLA_ (35.1 ± 47.7 spw, Figure 1B). As compared to the determined fold increase of the reference peptide A*03_EBNA3A_RLRA_, the long-term stimulation with one of the four highly immunodominant peptides resulted in higher rises of spw, reflected by average increases of 78.4- (A*03_BILF2_VTLA_), 71.4- (A*03_BcRF1_FLLA_), 30.4- (A*03_BALF3_QVAT_) and 44.8-fold (A*03_BILF2_LIIP_, Figure 1C). In short, the high immunodominance of the peptides A*03_BILF2_VTLA_, A*03_BcRF1_FLLA_, A*03_BALF3_QVAT_ and A*03_BILF2_LIIP_ was confirmed by strong IFN-γ secretion levels after long-term stimulation.

#### Increase of IFN-γ response in comparison to the mere EBV_Consensus pool

Addition of the four highly immunodominant peptides to the well-established peptide pool EBV_Consensus (Table 1, EBV_Consensus+4P_MIX_) yielded increased numbers of detectable EBV-specific T cells following both short- and long-term *in vitro* stimulation in comparison to the stimulation with the EBV_Consensus alone (Figure 1D). Short-term *in vitro* stimulation with EBV_Consensus+4P_MIX_ led to a 11.5 higher mean value of spw than stimulation with the mere EBV_Consensus and to 5.94% more spw per 1000 CD3^+^ T cells (data not shown).

#### Proof of immunodominance by detection of cytotoxicity and cytokine secretion

**Figure 2:**
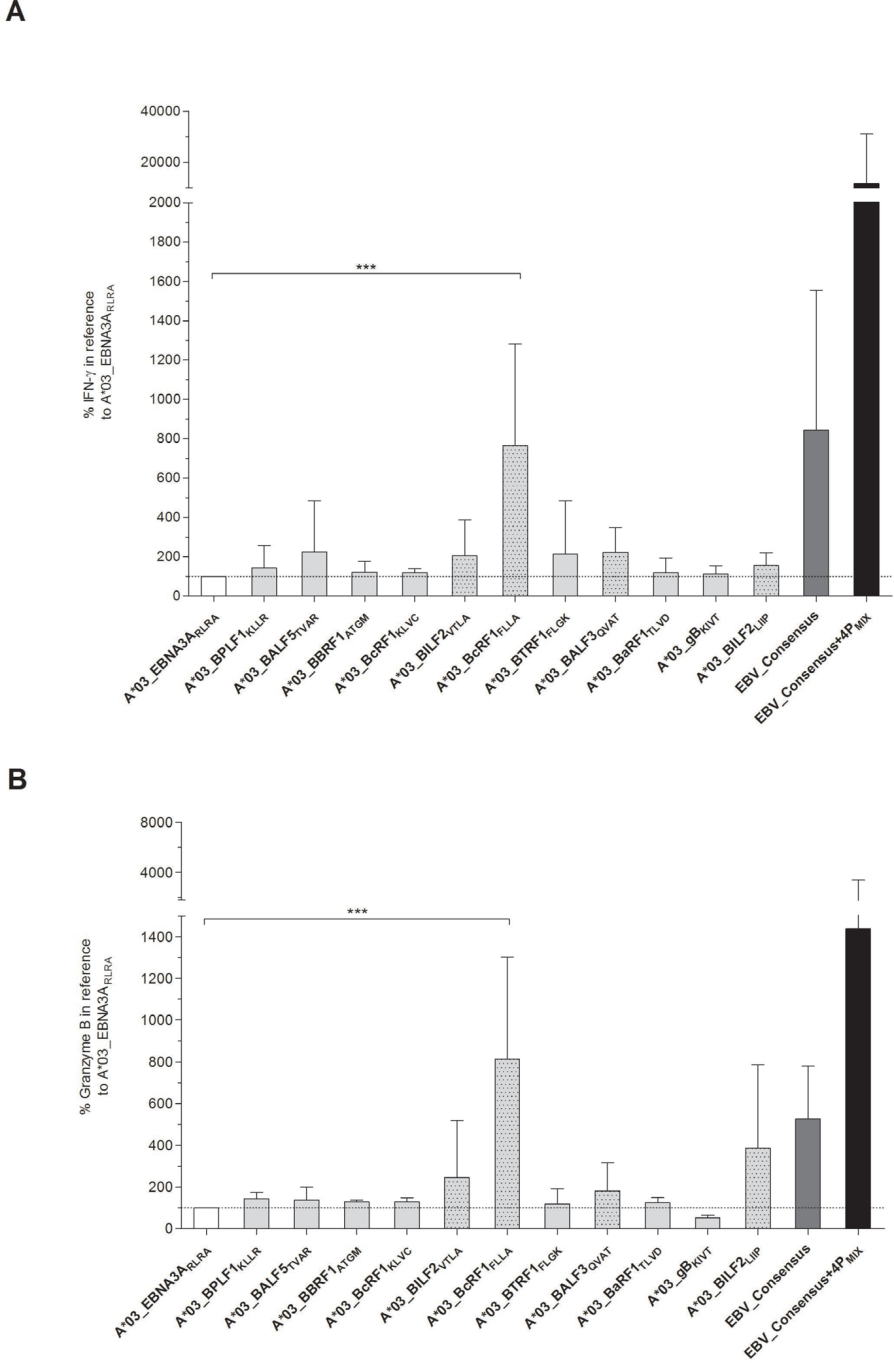
(**A**–**B**) Quantification of IFN-γ and granzyme B secretion by ELISA. Secretion of IFN-γ and granzyme B in response to the stimulation with one of the respective peptides was analyzed by quantitative ELISA after *in vitro* antigen-specific stimulation over seven days (*n* = 5). The referential antigens (A*03_EBNA3A_RLRA_, EBV_Consensus) were additionally employed as antigenic stimuli. Results of (A) IFN-γ and (B) granzyme B secretion (pg/ml) are shown for each of the peptides and EBV_Consensus+4P_MIX_ as the percentage concentration in reference to the peptide A*03_EBNA3A_RLRA,_ which is set to a comparative value of 100%. They are displayed as means ± SD. Asterisks indicate statistically significant differences between the EBV-specific peptides and A*03_EBNA3A_RLRA_ (^***^p < 0.001).

Apart from evaluating the immunogenicity by detection of IFN-γ secretion, the cytotoxic functionality of the EBV-specific peptide-induced T cells was assessed. After 7 days of *in vitro* stimulation with one of the eleven EBV-derived candidate-peptides, secretion levels of IFN-γ (Figure 2A) and granzyme B (Figure 2B) in supernatants were therefore determined. Secretion levels were comparable or even higher in comparison to the reference peptide A*03_EBNA3A_RLRA_ (IFN-γ: 1671 ± 2802 pg/ml; granzyme B: 3209 ± 2510 pg/ml, data not shown). The IFN-γ secretion levels in response to the newly identified immunodominant peptides were visualized as percentages in correlation to those of the reference peptide A*03_EBNA3A_RLRA_, which were set to a comparative value of 100%. They ranged from 113.2 ± 41.2% (A*03_gB_KIVT_) to 765.2 ± 516.5% (A*03_BcRF1_FLLA_, Figure 2A). The granzyme B secretion levels varied between 52.9 ± 12.2% (A*03_gB_KIVT_) and 813.4 ± 489.6% (A*03_BcRF1_FLLA_, Figure 2B). Thus, the peptide A*03_BcRF1_FLLA_ consistently induced the highest secretion of IFN-γ and of granzyme B, which were significantly higher than those induced by the reference peptide (^***^p < 0.001). The stimulating efficacy of the mixture EBV_Consensus+4P_MIX_ compared to the EBV_Consensus revealed a 13.8-fold higher IFN-γ secretion (EBV_Consensus: 844.0% versus EBV_Consensus+4P_MIX_: 11642.0%, Figure 2A). Regarding granzyme B, the stimulation with EBV_Consensus+4P_MIX_ led to a 2.73-fold higher concentration than the stimulation with the mere peptide pool (EBV_Consensus: 526.2% versus EBV_Consensus+4P_MIX_: 1439.0%, Figure 2B).

#### IFN-γ/granzyme B FluoroSpot assay to confirm the high immunodominance of four of the EBV-derived peptides as single stimulants and in combination

The immunogenic and cytotoxic functionality of the four newly identified highly immunodominant peptides was further affirmed by IFN-γ/granzyme B FluoroSpot assay (Supplementary Figure 2). The highest values for IFN-γ producing cells were detected with A*03_BcRF1_FLLA_ (1.70 ± 1.48 spw) and A*03_BALF3_QVAT_ (1.69 ± 1.79 spw, Supplementary Figure 2). The stimulation with the reference peptide A*03_EBNA3A_RLRA_ however led on average to a 1.55-fold higher amount of IFN-γ producing cells (2.60 ± 3.27 spw). With respect to the cytotoxic T-cell activity, A*03_EBNA3A_RLRA_ induced on average a 0.59-fold lower number of granzyme B producing cells (0.70 ± 1.10 spw) than A*03_BcRF1_FLLA_ (0.80 ± 1.79 spw) and A*03_BALF3_QVAT_ (1.57 ± 3.32 spw, Supplementary Figure 2). Similar to the respective numbers of IFN-γ producing cells, the stimulation with A*03_BILF2_VTLA_ and A*03_BILF2_LIIP_ resulted in low numbers of granzyme B producing cells.

**Figure 3:**
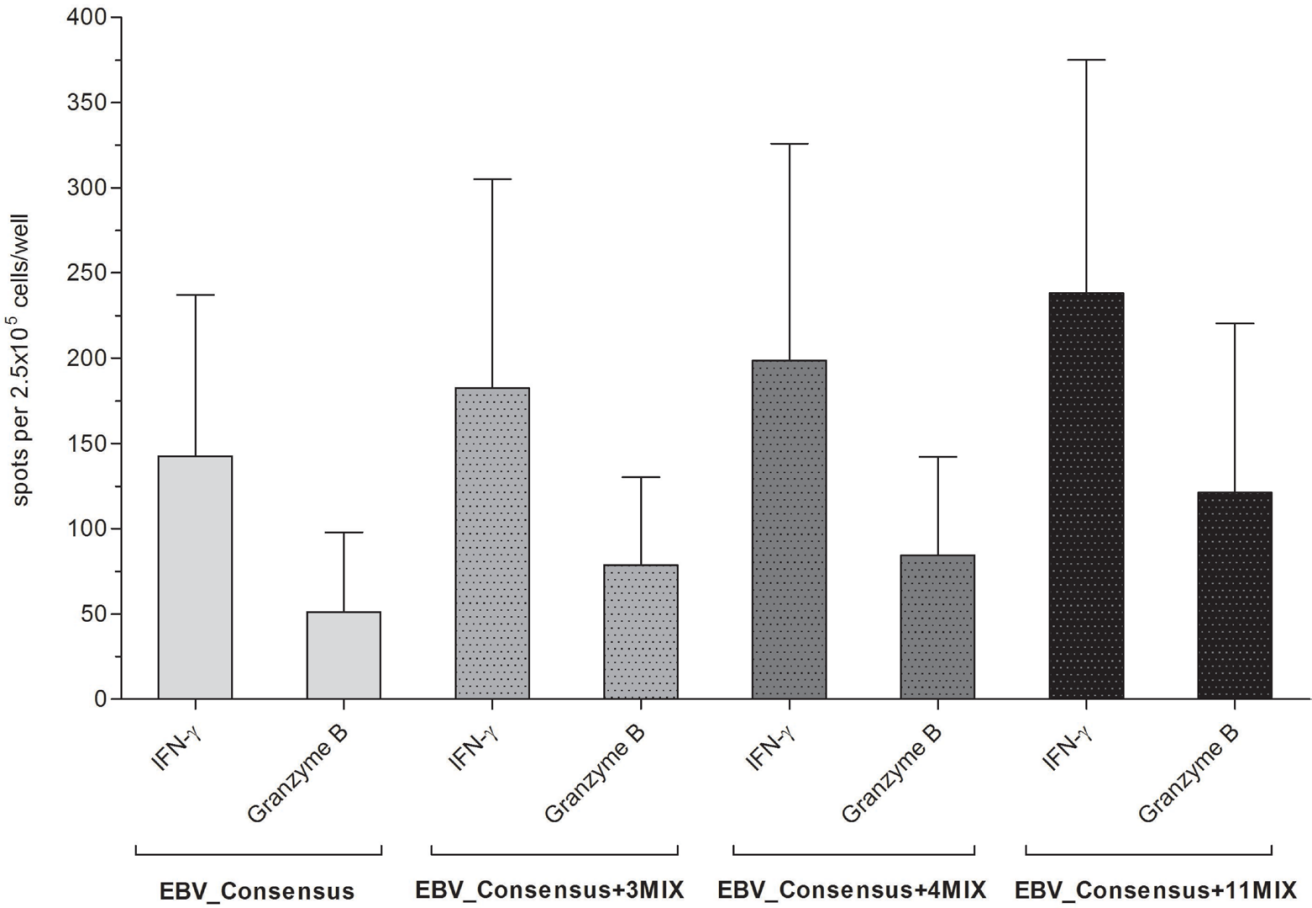
Evaluation of the immunogenic and cytotoxic activity of the peptide-induced EBV-specific T cells. The cytotoxic activity of peptide-induced EBV-specific T cells was investigated by means of IFN-γ and granzyme B FluoroSpot assays. PBMCs from healthy HLA-A*03:01-positive, EBV-seropositive donors (*n* = 5) were stimulated with one of the three peptide-mixtures (EBV_Consensus+3P_MIX_, EBV_Consensus+4P_MIX_, EBV_Consensus+11P_MIX_, Table 1). The mere EBV_Consensus served as referential antigen. Spots identified with the filter for FITC represented IFN-γ producing cells and spots identified by the filter for Cy3 detected granzyme B producing cells. Results are given as the number of spots per well (spw), representing the number of spots in the antigen well after subtracting those of the respective negative control well. They are displayed as means ± SD.

The stimulation with one of the three applied mixtures EBV_Consensus+3P_MIX_, EBV_Consensus+4P_MIX_ and EBV_Consensus+11P_MIX_ triggered the secretion of both IFN-γ and granzyme B (Figure 3). The results were in line with the higher stimulating efficacy obtained via IFN-γ EliSpot assays and demonstrated a step-by-step enhancement of 28.1% (182.6 ± 122.4 spw, EBV_Consensus+3P_MIX_), 39.3% (198.6 ± 127.4 spw, EBV_Consensus+4P_MIX_) and 67.0% (238.2 ± 136.9 spw, EBV_Consensus+11P_MIX_), as compared to the IFN-γ producing cells induced by EBV_Consensus (142.6 ± 94.5 spw, Figure 3). Correspondingly, this stepwise enhancement was determined in regard to the cytotoxic T-cell activity mirrored by the quantification of granzyme B producing cells, unveiling an enhancement of 53.6% (78.6 ± 51.8 spw, EBV_Consensus+3P_MIX_), 64.8% (84.4 ± 57.8 spw, EBV_Consensus+4P_MIX_) and 136.7% (121.2 ± 99.2 spw, EBV_Consensus+11P_MIX_) in comparison to the stimulation with just the EBV_Consensus (51.2 ± 46.6 spw, Figure 3).

The obtained results confirmed the immunodominance of the eleven EBV-specific T-cell epitopes, showed that the stimulation capacity of an established peptide pool can be improved and underlined the classification of the four peptides A*03_BILF2_VTLA_, A*03_BcRF1_FLLA_, A*03_BALF3_QVAT_ and A*03_BILF2_LIIP_ as highly immunodominant.

### Confirmation of the EBV-derived peptides’ *in vitro* binding and dissociation

**Figure 4:**
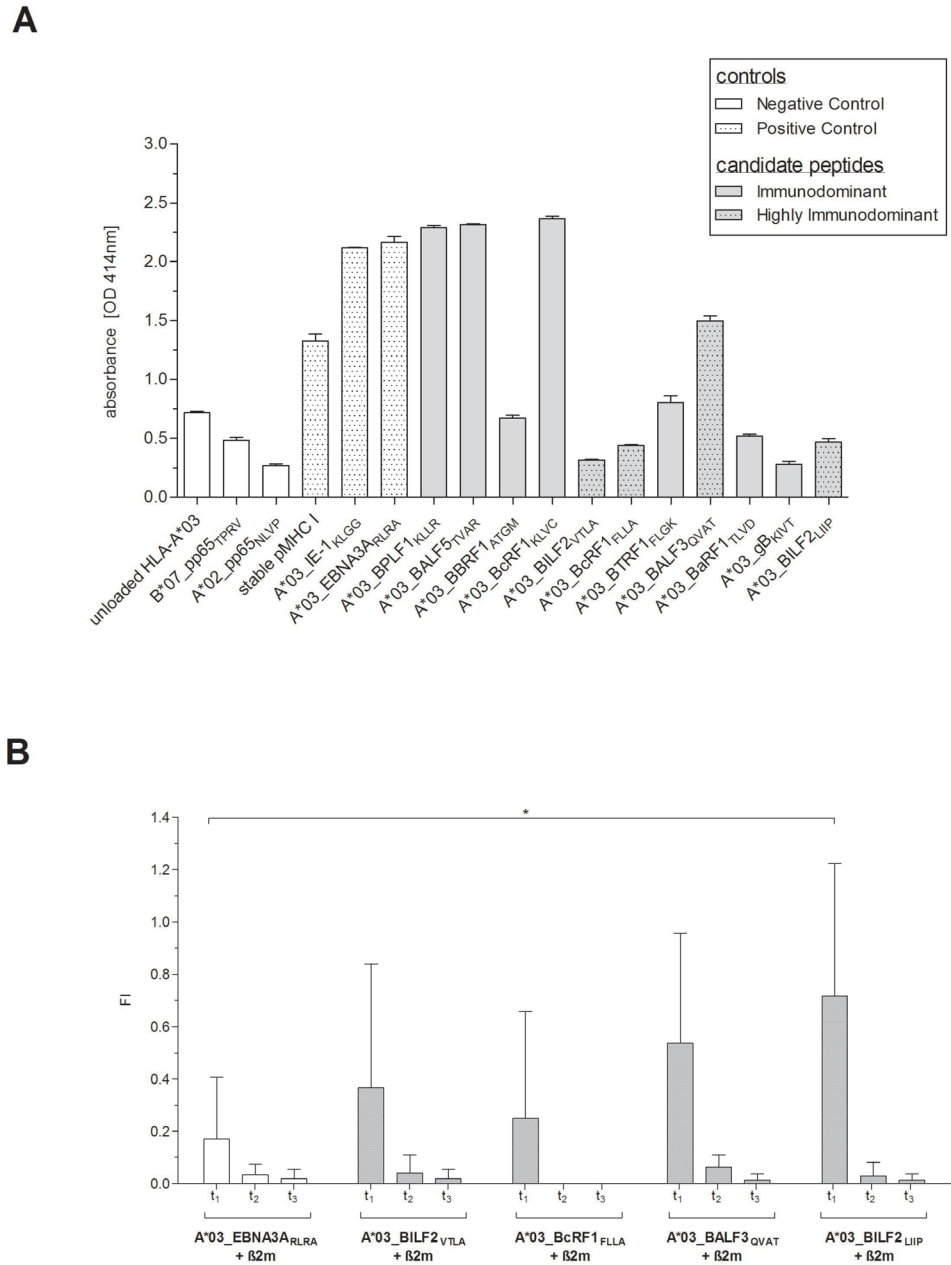
(**A**–**B**) Confirmation of the EBV-derived peptides’ *in vitro* binding and dissociation. Peptide-binding efficiency on HLA-A*03:01 monomers and stability of the pHLA-A*03:01-complexes of the newly identified EBV-derived candidate-peptides (Table 1) were determined by (A) Flex-T^TM^ MHC Tetramer assays (Flex-T assay) and by (B) T2 peptide binding and dissociation assays. Flex-T assays were carried out for all eleven candidate-peptides. A stable HLA class I monomer with a high-affinity, non UV-labile peptide as well as pMHC monomers for the known HLA-A*03:01-restricted CMV-IE1-specific peptide KLGGALQAK (A*03_IE1_KLGG_) and A*03_EBNA3A_RLRA_ (Table 1) functioned as positive controls. Unloaded HLA-A*03:01 monomers (unloaded HLA-A*03:01) as well as pMHC monomers for the CMVpp65-derived peptides restricted to HLA-A*02:02 (NLVPMVATV, A*02_pp65_NLVP_) and HLA-B*07:02 (TPRVTGGGAM, B*07_pp65_TPRV_), respectively, served as negative controls. (A) Efficiency of the pivotal exchange process was verified by a rapid streptavidin-capture ELISA and results are given as the optical density (OD), quantified at 414 nm absorbance. The stability of the pHLA-A*03:01-complexes in response to the four highly immunodominant EBV-derived peptides was investigated by T2 peptide binding and dissociation assays. Peptide-unloaded T2 cells served as negative control and the known immunodominant HLA-A*03:01-restricted EBV-derived peptide RLRAEAQVK (A*03_EBNA3A_RLRA_) as reference. (B) The HLA class I expression levels as well as the peptide-MHC-complex dissociation were investigated at the given points of time (t_1_ = 0 min, t_2_ = 60 min, t_3_ = 120 min). The resultant fluorescence index (FI) was calculated as the mean fluorescence intensity (MFI) of HLA-A*03:01 on peptide-stimulated transduced T2 cells and peptide-unloaded cells, respectively. In terms of a comparable reference the unloaded cells were standardized to 0. The results are shown as the mean of *n* = 3 independent experiments **±** SD and statistically significant differences between the EBV-specific peptides and A*03_EBNA3A_RLRA_ are asterisked (^*^p < 0.05).

To confirm the *in vitro* peptide binding and the stability of the peptide-HLA-A*03:01 (pHLA-A*03:01)-complexes, Flex-T and T2 peptide binding and dissociation assays were performed. The peptide-MHC (pMHC)-binding capacity of all eleven newly identified peptides to HLA-A*03:01 monomers was assessed using the Flex-T assay (Figure 4A). The three peptides, A*03_BPLF1_KLLR_ (2.29 OD_414_), A*03_BALF5_TVAR_ (2.32 OD_414_) and A*03_BcRF1_KLVC_ (2.37 OD_414_), all of them predicted to be either strong or weak binders (Table 1), revealed strong binding to HLA-A*03:01 as shown by the highest optical densities (OD). The other candidates displayed ODs in the range of the negative control peptides. The lowest absorbance was detected for the A*03_gB_KIVT_-specific monomer (0.28 OD_414_), predicted to be a weak binder. The ELISAs for three of the four peptides classified as highly immunodominant also resulted in low absorbance values (0.32–0.47 OD_414_).

Peptide-binding and stability of the pHLA-A*03:01-complexes of the four highly immunodominant candidate-peptides were furthermore investigated by T2 peptide binding and dissociation assay (Figure 4B). Peptide binding as indicated by the first time point (t_1_) was higher for all four peptides than for the reference peptide A*03_EBNA3A_RLRA_ (0.17 ± 0.24 FI) with fluorescence indexes (FIs) ranging from 0.25 ± 0.41 (A*03_BcRF1_FLLA_) to 0.72 ± 0.51 (A*03_BILF2_LIIP_, *n* = 3, Figure 4B). The highest binding capacity was detected for A*03_BILF2_LIIP_ revealing a 4.22-fold higher FI than A*03_EBNA3A_RLRA_. Interestingly, the predictions of both the peptide-binding affinity (Table 1) and the half-life for A*03_BILF2_LIIP_ were however the lowest of all these eleven newly identified EBV-derived peptides. After 60 minutes (t_2_) the FI values for the four pMHC complexes were on average 93.3% lower than those quantified at t_1_. A*03_BcRF1_FLLA_ unveiled the fastest dissociation, since no FI value was detectable after 60 minutes (t_2_), which corresponded to the predicted short half-life of 0.55 hours (NetMHCstab, www.cbs.dtu.dk/services/NetMHCstab-1.0/, data not shown). A step-by-step dissociation was observed for A*03_BILF2_VTLA_ and A*03_BILF2_LIIP_ with FI values at t_2_ being 89.1% and 95.8% lower, respectively, than at t_1_. The highest FI value at t_2_ was detected in response to A*03_BALF3_QVAT_ (Figure 4B), thus in line with the predicted half-life of 2.57 hours (NetMHCstab, www.cbs.dtu.dk/services/NetMHCstab-1.0, data not shown). Although the FI values of t_1_ for all four peptides decreased by on average 97.6% after 120 minutes (t_3_, Figure 4B), a certain number of peptide-specific pMHC complexes, as mirrored by the FI values, revealed to be stable and was thus detectable.

Comparing the prediction scores to the results of both the Flex-T assay and the *in vitro* T2 dissociation assay, the reference peptide A*03_EBNA3A_RLRA_ revealed the highest pMHC complex stability, followed by the candidate-peptide A*03_BALF3_QVAT_. The peptide-binding capacity of the four highly immunodominant peptides was confirmed by the HLA class I binding and dissociation assay.

### Peptide-specific T cells detectable in peripheral blood of healthy donors without prior stimulation

**Figure 5:**
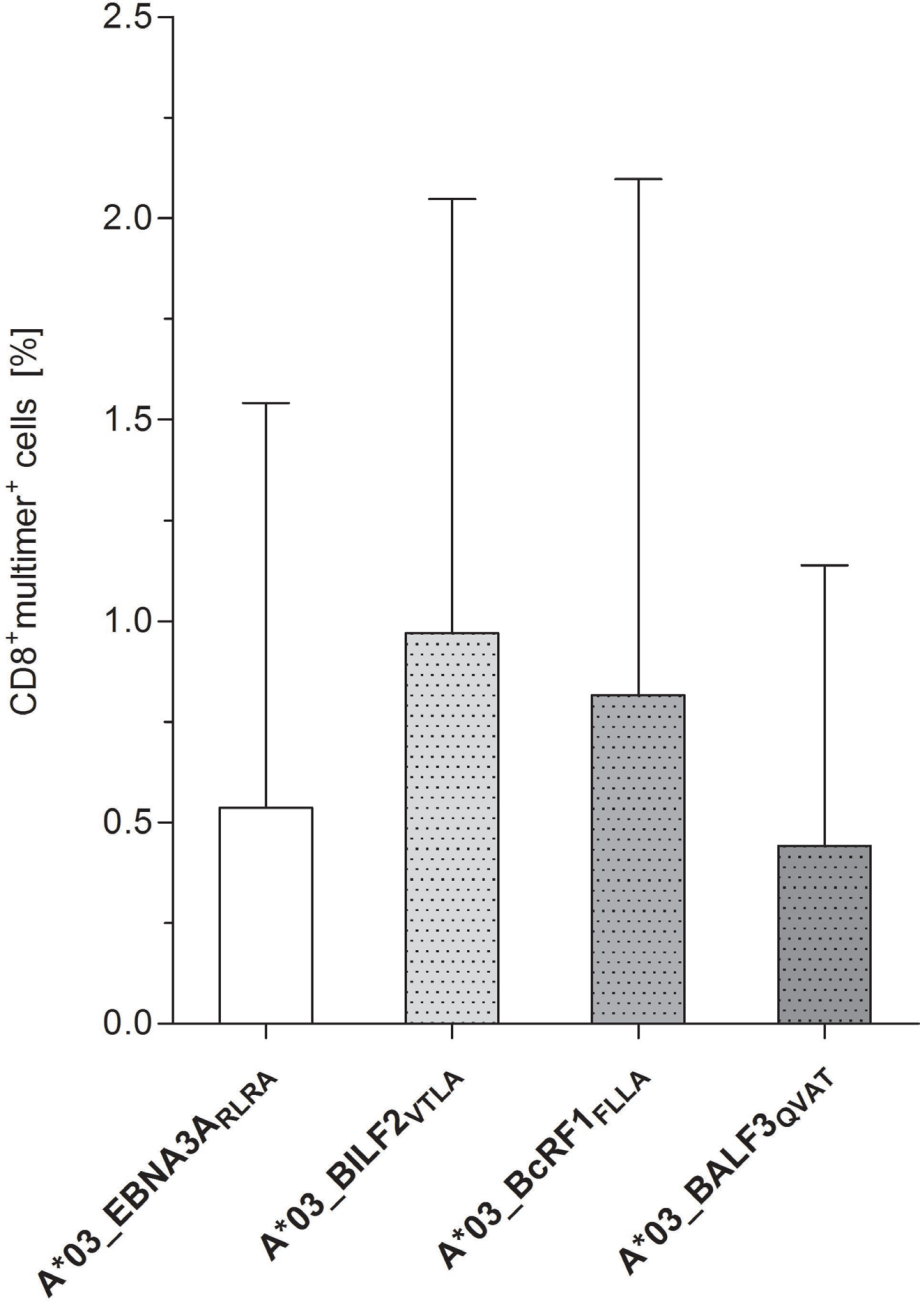
Identification of peptide-specific CTL precursor frequencies in healthy donors by pMHC multimer staining. Precursor frequencies of EBV-specific CTLs directed against the peptides A*03_BILF2_VTLA_, A*03_BcRF1_FLLA_ and A*03_BALF3_QVAT_ were visualized via pMHC multimer staining, analyzed by flow cytometry and compared to the frequencies of the referential antigen A*03_EBNA3A_RLRA_ of freshly isolated PBMCs (day 0, *n* = 5). Findings are displayed as the mean percentage of CD8^+^multimer^+^ T cells **±** SD after subtracting the respective percent value of the general nonsense dextramer, which functioned as a negative control.

The precursor frequencies of circulating CD8^+^ CTLs specific to the newly identified, highly immunodominant EBV-derived epitopes A*03_BILF_VTLA_, A*03_BcRF1_FLLA_ and A*03_BALF3_QVAT_ were determined by multimer staining in freshly isolated PBMCs of healthy donors (Figure 5, Supplementary Figure 4). In comparison to the reference peptide A*03_EBNA3A_RLRA_ (0.54%, range: 0–2.31%), the detectable peptide-specific CTLs reached frequencies ranging from 0.44 ± 0.70 to 0.97 ± 1.08% (Figure 5). The frequencies of circulating CTLs specific to A*03_BALF3_QVAT_ (0.44% range: 0–1.66%) were lower than those specific to A*03_BILF2_VTLA_ (0.97%, range: 0.15–2.82%) and A*03_BcRF1_FLLA_ (0.82%, range: 0.02–3.09%, Figure 5). T-cell responses against A*03_BILF2_VTLA_ (*n* = 3/5), A*03_BcRF1_FLLA_ (*n* = 3/5) and A*03_EBNA3A_RLRA_ (*n* = 2/5) as detected by multimer staining were consistent with the corresponding IFN-γ EliSpot results. Regarding the peptide A*03_BALF3_QVAT_, a lower number of positive donors (*n* = 2/5) was identified by multimer staining in comparison to IFN-γ EliSpot (Figure 1B).

### Efficient enrichment of EBV-specific CTLs induced by the newly identified highly immunodominant CTL epitopes

#### Enriching the IFN-γ secreting T cells via stimulation with the single peptides

**Figure 6:**
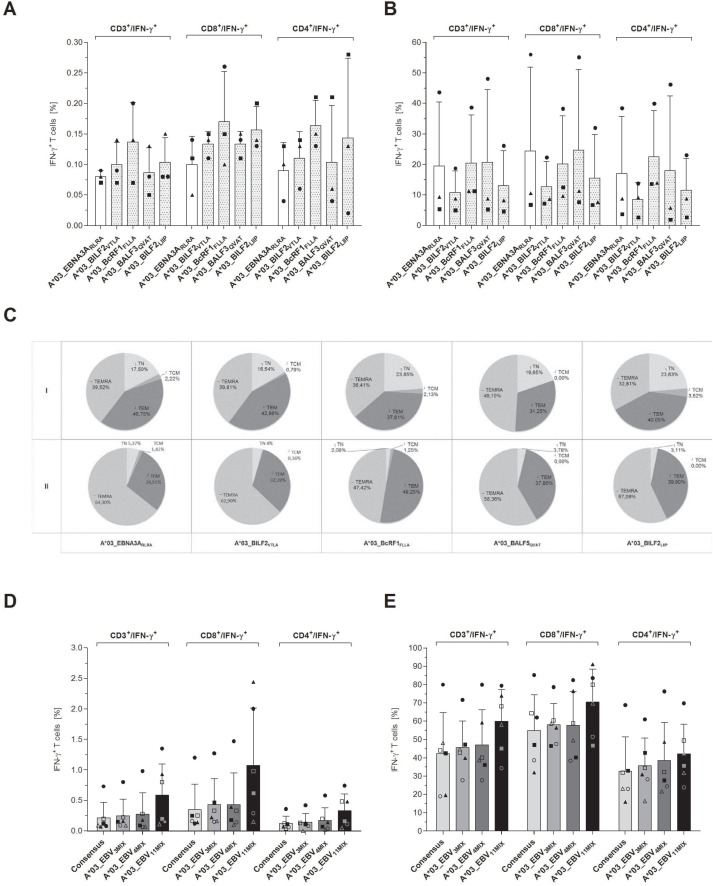
(**A**–**E**) Evaluation of the eligibility of the highly immunodominant HLA-A*03-restricted EBV-derived peptides for clinical application–by means of CSA. PBMCs from healthy donors (*n* = 3) were stimulated with one of the four highly immunodominant EBV-peptides. Respective frequencies of the IFN-γ-secreting cell fractions before (origin) and after the enrichment (eluate) were determined by multicolor flow cytometry. Frequencies in the ‘origin’ evaluated in comparison to (A) the respective frequencies of the IFN-γ-secreting T cells in response to the EBV-derived reference peptide A*03_EBNA3A_RLRA_. (B) The efficiency of the IFN-γ-specific enrichment via the magnetic labeling of IFN-γ-secreting cells is separately shown for each of the assessed peptides. (C) Phenotypic analyses regarding naïve (T_N_), central memory (T_CM_), effector memory (T_EM_) and terminally differentiated effector memory (T_EMRA_) T cells were performed for the IFN-γ^+^CD8^+^-secreting T cells of both aliquots (I: Origin, II: Eluate) and are visualized by the respective mean frequencies. To determine the peptides’ aptitude to enhance the stimulating efficacy of the EBV_Consensus, three mixtures of the *in vivo* isolated peptides and the EBV_Consensus were used as stimulating antigens (EBV_Consensus+3P_MIX_, EBV_Consensus+4P_MIX_, EBV_Consensus+11P_MIX_, Table 1). The frequencies of the different cell fractions in the ‘origin’ (*n* = 6) are individually displayed (donor 1 = ●, donor 2 = ■, donor 3 = ▲, donor 4 = ○, donor 5 = □ donor 6 = Δ) in comparison to the respective percent values of the (D) IFN-γ^+^CD3^+^-secreting T cells, IFN-γ^+^CD8^+^-secreting T cells and IFN-γ^+^CD4^+^-secreting T cells, respectively, induced by the mere EBV_Consensus. The efficiency of the enrichment is furthermore individually shown for each of the donors (*n* = 6) using the percent values of (E) the IFN-γ^+^CD3^+^-secreting T cells, the IFN-γ^+^CD8^+^-secreting T cells and the IFN-γ^+^CD4^+^-secreting T cells. Findings are displayed as individual results and as the mean percentage of IFN-γ^+^ T cells **±** SD.

IFN-γ cytokine secretion assay (CSA) was further on performed to evaluate the *in vivo* identified peptides as suitable target antigens for the generation of clinical-grade EBV-specific T cells (Figure 6). All four investigated candidate-peptides triggered on average 33.4% higher numbers of IFN-γ-producing CD3^+^ T cells in the origin fraction than the reference peptide A*03_EBNA3A_RLRA_ (A*03_EBNA3A_RLRA_: 0.08 ± 0.01%, A*03_BILF2_VTLA_: 0.10 ± 0.04%, A*03_BcRF1_FLLA_: 0.14 ± 0.07%, A*03_BALF3_QVAT_: 0.09 ± 0.04%, A*03_BILF2_LIIP_: 0.10 ± 0.04%, Figure 6A). The highest frequencies of IFN-γ^+^CD8^+^ T cells were observed for A*03_BcRF1_FLLA_ (0.17 ± 0.08%). The total number of IFN-γ^+^CD3^+^ T cells in the origin fraction is shown in Supplementary Figure 3A.

The enrichment process led to frequencies of IFN-γ-secreting CD3^+^ T cells ranging from 10.8 ± 7.11% (A*03_BILF2_VTLA_) to 20.7 ± 23.7% (A*03_BALF3_QVAT_), comparable to the enrichment efficiency of A*03_EBNA3A_RLRA_ (19.4 ± 21.0%, Figure 6B). Concerning the enrichment of IFN-γ^+^CD8^+^ T cells, increases ranged on average from 95.0- (A*03_BILF2_VTLA_) to 184.9-fold (A*03_BALF3_QVAT_). The enrichment of A*03_EBNA3A_RLRA_-specific IFN-γ^+^CD8^+^ T cells resulted in increases of 244.0-fold (Figure 6A and 6B).

The IFN-γ^+^CD8^+^ T cells were furthermore characterized by immunophenotyping. High proportions of T_EM_ (37.9 ± 4.95%) and T_EMRA_ (39.5 ± 6.99%) were assessed for each peptide before enrichment (Figure 6C). In comparison to T_EMRA_, slightly higher T_EM_ proportions were found in response to A*03_BILF2_VTLA_, A*03_BcRF1_FLLA_ and A*03_BILF2_LIIP_ with differences of 7.12%, 3.20% and 18.1% (Figure 6C). The stimulation with A*03_BALF3_QVAT_ however led to a 36.4% higher T_EMRA_ proportion in relation to T_EM_. Similar frequencies of T_EM_ (40.8%) and T_EMRA_ (39.5%) were detected following stimulation with the reference peptide A*03_EBNA3A_RLRA_. Regarding T_CM_ proportions, frequencies ranged from 0% (A*03_BALF3_QVAT_) to 3.52% (A*03_BILF2_LIIP_). The comparative analysis of the CD45RA^+^ and of the CD45RA^-^ frequencies showed on average a higher proportion of CD45RA^+^ T cells (60.4%) and a 20.9% lower frequency of CD45RA^-^ T cells (39.6%, Figure 6C).

#### Improving the enrichment efficacy of EBV-specific T cells using peptide mixtures

To confirm the suitability of the newly identified peptides for the generation of clinical-grade T cells, the additional antigen-specific stimulation with the three different mixtures was evaluated in comparison to the EBV_Consensus alone (Figure 6D–6E). The stimulation with EBV_Consensus+3P_MIX_ resulted in the activation of IFN-γ^+^CD3^+^ T cells with a mean frequency of 0.25 ± 0.27% (Figure 6D), which was 17.2% higher in comparison to the mere EBV_Consensus (EBV_Consensus: 0.22 ± 0.26%, Figure 6D). The highest capacity was homogenously detected in all donors subsequent to a stimulation with EBV_Consensus+11P_MIX_. The stimulation with this mixture led to 174.4% more IFN-γ^+^CD3^+^ T cells (0.59 ± 0.51%, Figure 6D) and 205.9% more IFN-γ^+^CD8^+^ T cells (1.08 ± 0.94%, Figure 6D), as compared to the mere EBV_Consensus.

The proportions of IFN-γ^+^CD3^+^ T cells following enrichment revealed a donor-specific enrichment efficiency (Figure 6E). With respect to the different antigenic stimulations, the enrichment process in donor 3 demonstrated a homogenous step-by-step enhancement of IFN-γ^+^CD3^+^ T cells. In donor 1 and 2, however, this process did not induce any higher proportions of IFN-γ^+^CD3^+^ T cells than the EBV_Consensus alone (Figure 6E). Taking the immunophenotypes of the IFN-γ^+^CD8^+^ T cells into account before and after the enrichment process, it becomes evident that the stimulation with one of these three mixtures triggered higher T_EM_ (45.4 ± 3.44% T_EM_ (Origin), 63.4 ± 4.75% T_EM_ (Elution)) than T_EMRA_ proportions (33.8 ± 1.13% T_EMRA_ (Origin), 28.8 ± 4.89% T_EMRA_ (Elution)). The mean frequency of T_CM_ ranged from 5.21 ± 4.87% to 10.2 ± 9.41% prior to the enrichment (data not shown). In summary, these findings highlight the suitability of the newly identified epitopes as antigenic stimulants for the generation of clinically applicable EBV-specific T cells.

### Clinical relevance of the newly identified highly immunodominant CTL epitopes in patients with EBV-associated PTLD

**Figure 7:**
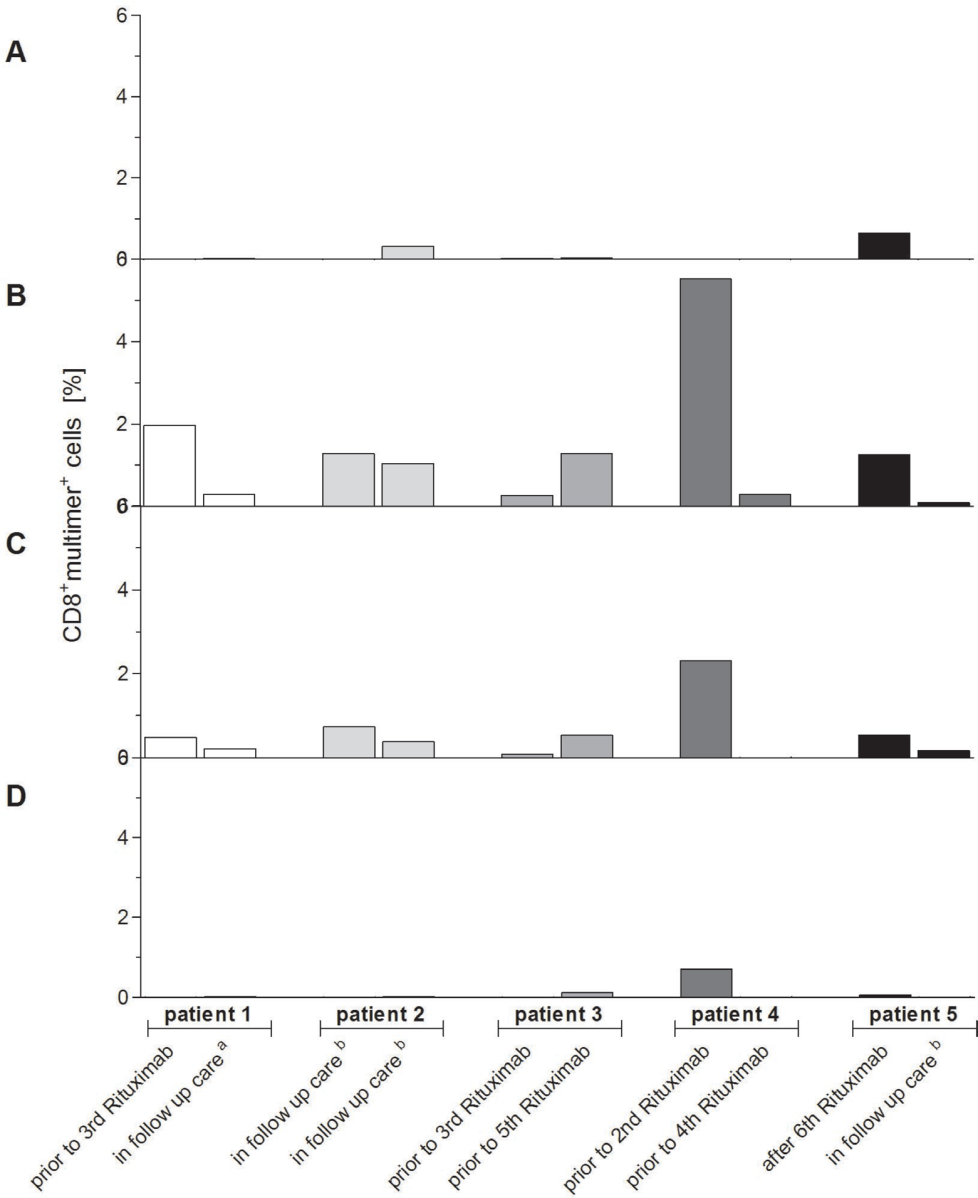
(**A**–**D**) Verification of clinical relevance of the newly identified highly immunodominant CTL epitopes for EBV-associated PTLD. The clinical relevance of the highly immunodominant EBV-derived peptides (A*03_BILF2_VTLA_, A*03_BcRF1_FLLA_, A*03_BALF3_QVAT_) and the known immunodominant EBV-derived peptide (A*03_EBNA3A_RLRA_) for EBV-associated PTLD was verified by pMHC multimer staining and analyzed by multicolor flow cytometry. The respective frequencies of peptide-specific CD8^+^multimer^+^ T cells in HLA-A*03:01-positive patients suffering from EBV-associated PTLD were visualized by separately displaying the detected frequencies at two different points of time in the course of their treatment (*n* = 5). In order to exclude false-positive cells due to unspecific background level the general nonsense was applied and its respective percent value was subtracted from the patients’ frequencies specific to one of the peptides. At the time of blood withdrawal patients were prior to receiving their second, third, fourth or fifth treatment with rituximab, after their sixth treatment with rituximab, in follow-up care ensuing cytotoxic chemotherapy (^a^) or in follow-up care subsequent to no other treatment than rituximab (^b^). The resultant precursor frequencies of peptide-specific CD8^+^multimer^+^ T cells are shown as follows: (A) A*03_EBNA3A_RLRA_, (B) A*03_BILF2_VTLA_, (C) A*03_BcRF1_FLLA_ and (D) A*03_BALF3_QVAT_.

The relevance and clinical applicability of the highly immunodominant peptides A*03_BILF2_VTLA_, A*03_BcRF1_FLLA_ and A*03_BALF3_QVAT_ for PTLD and EBV-associated diseases were verified in HLA-A*03:01^+^ patients with EBV-associated PTLD after SOT. Peptide-specific T-cell frequencies differed during the patients’ treatment (Figure 7A–7D, Supplementary Figure 4). A*03_EBNA3A_RLRA_-specific CTLs were not detectable (Figure 7A) in all patients, whereas for A*03_BILF2_VTLA_ (Figure 7B) and A*03_BcRF1_FLLA_ (Figure 7C) well-defined peptide-specific CD8^+^multimer^+^ populations were detected in each of the assessed patients. For the A*03_BALF2_QVAT_ epitope (Figure 7D) well-defined CD8^+^multimer^+^ populations were found in only 40% of the patients. The multimer staining resulted in the detection of on average 1.33% A*03_BILF2_VTLA_-specific CTLs (range: 0.09–5.52%, Figure 7B) and 0.52% A*03_BcRF1_FLLA_-specific CTLs (range: 0–2.31%, Figure 7C). Both revealed on average higher frequencies in the patients’ blood than the applied reference A*03_EBNA3A_RLRA_ (0.03% within CD8^+^ T cells, range: 0–0.65%, Figure 7A). These higher frequencies of A*03_BILF2_VTLA_- and A*03_BcRF1_FLLA_-specific CTLs were in line with those of the peptide-specific CTLs detected in the assessed healthy donors (Figure 5), while the lowest mean frequency was discerned for A*03_BALF3_QVAT_ (0.004%, range: 0–0.71%, Figure 7D).

Interestingly, the highest frequencies for A*03_BILF2_VTLA_ (5.52%, Figure 7B), A*03_BcRF1_FLLA_ (2.31%, Figure 7C) and A*03_BALF3_QVAT_ (0.71%, Figure 7D) were detected in the blood of patient 4 prior to the second rituximab therapy. Before administering the fourth rituximab dose to patient 4, solely frequencies of A*03_BILF2_VTLA_-specific CTLs continued to be discernible (0.30%, Figure 7B) in contrast to A*03_BcRF_FLLA_ (Figure 7C) and A*03_BALF3_QVAT_ (Figure 7D).

Concisely, EBV-specific T cells of differing frequencies were detectable in response to all investigated peptides in patients with PTLD. These differing frequencies underline the importance of knowing a broad spectrum of pathogen-specific T-cell epitopes, which would enable the monitoring of specific T-cell frequencies.

## SUMMARY

In this study, eleven novel EBV-specific CTL epitopes deriving from nine different EBV-proteins were isolated *in vivo* and selected by epitope prediction tools. All eleven CTL epitopes were identified as immunodominant by strong cytokine secretion and cytotoxicity. The four peptides A*03_BILF2_VTLA_, A*03_BcRF1_FLLA_, A*03_BALF3_QVAT_ and A*03_BILF2_LIIP_ were classified as highly immunodominant. The clinical relevance was verified by detection of the respective antiviral CD8^+^ T-cell frequencies specific to three of these epitopes in patients with EBV-associated PTLD. The eligibility for adoptive immunotherapy was confirmed regarding the four highly immunodominant CTL epitopes by CSA, when being applied as single stimulants and in combination with an already established peptide pool.

## DISCUSSION

To improve the clinical outcome of patients suffering from EBV-associated post-transplant complications the present study aimed at the identification of novel, naturally presented and thus *in vivo* relevant EBV-derived T-cell epitopes suitable to (1) monitor EBV-specific immunity and to (2) generate highly efficient and clinically relevant T-cell products. The given experimental approach is a proof of concept with well-suited eligibility for the identification of novel epitopes restricted to specific HLA alleles, potentially applicable to further HLA alleles and/or target antigens.

### Adoptive immunotherapy with EBV-specific T cells: Advantageous potential of peptides and their limitations

Adoptive immunotherapy with EBV-specific T cells provides a personalized, targeted and non-toxic immunotherapeutic strategy for the immediate and long-term protection of the patients’ EBV-specific immunity. This promising therapeutic approach is however limited by the high antigenic diversity due to distinct, latency-associated gene and protein expression profiles, the HLA-restrictions as well as limited by the low immunodominance of known EBV epitopes [24]. Icheva *et al.* demonstrated that the adoptive transfer of EBNA1-specific T cells to patients with EBV viremia and/or PTLD after HSCT is safe as neither GvHD (>grade 2) nor acute toxicity have been detected [13]. Since EBNA1 is the EBV protein to be expressed in all three latency states and relevant to EBV-associated PTLD, it represents one of the most promising antigenic targets [55]. In 80% of the patients, *in vivo* expansion of EBNA1-specific T cells could be observed and in 70% of them clinical and virological responses were detected, whereas 30% of the PTLD patients did not reveal any kind of effective immune response [13]. These findings highlight the fact that known EBNA1-derived epitopes are of low immunogenicity, thus leading to low frequencies and weak cytotoxic T-cell responses [47, 55, 56]. So far, this impedes the therapeutic efficacy of adoptive immunotherapy for EBV-associated diseases in immunocompromised patients.

The major challenge of developing and improving immunotherapeutic strategies is therefore to overcome these limitations. Mixtures of multiple immunogenic peptides instead of single peptides deriving from one or more EBV-specific proteins and covering multiple HLA class I and II alleles were found to be advantageous [1, 57] by inducing EBV-specific T cells of higher antigenic diversity, essential to an effective treatment of EBV-associated PTLD [17, 58]. Recently, Nowakowska *et al.* have detected an enhanced stimulating efficacy by the use of a mixture of peptides deriving from 16 latent and lytic EBV proteins in comparison to the stimulation with peptide pools covering just sequences of either EBNA1, LMP2A, BZLF1 or EBNA3C [58]. Consequently, a larger pool of antigens derived from various EBV proteins with different HLA restriction elements reveals to be more efficacious and leads to higher response rates resulting in the activation of a greater EBV-specific memory T-cells’ repertoire [17, 58].

Moosmann *et al.* rapidly generated and isolated EBV-specific T cells by using a pool of 23 EBV peptides deriving from 11 EBV proteins [17]. This peptide pool covered the most frequent, but not all HLA-class I and II alleles [17]. The commercially available peptide pool PepTivator EBV Consensus (Miltenyi Biotec, indicated as ‘Select’ in GMP grade), containing 43 peptides and deriving from 13 different lytic and latent EBV-proteins, is likewise restricted to 14 frequent HLA-class I and II molecules. Since the stimulation spectrum is exclusively narrowed to those alleles represented in these pools, even the latter are not irrespective of the donor's HLA-type. This emphasizes the necessary enlargement of the repertoire of T-cell epitopes restricted to certain, currently underrepresented HLA-alleles [47]. For validation of the hereby applied experimental approach to broaden the HLA-coverage of these overlapping EBV-specific peptide pools, the given study focused on the HLA-A*03:01-allele, as only few EBV-specific HLA-A*03:01-restricted epitopes have been identified, only two epitopes restricted to HLA-A*03:01 have been commercially available (EBNA3A: RLRAEAQVK, BRLF1: RVRAYTYSK, [47]) and have therefore been primarily used as HLA-A*03:01-restricted stimuli (e.g. [17, 58]).

### Highly immunotherapeutic potential of the newly identified EBV-derived peptides

EBV-specific peptides were isolated *in vivo* from deliberately chosen EBV-infected sHLA-A*03:01-transduced B-LCLs, expressing all ten EBV-latency-associated proteins of latency state III and therefore representing a surrogate for PTLD [5, 55]. This was done to guarantee the clinical relevance for EBV-infections, -reactivations and EBV-associated PTLD and to ensure the *in vivo* natural presentation of the candidate-epitopes.

Eleven *in vivo* isolated EBV-specific HLA-A*03:01-restricted T-cell epitopes were identified as potential targets by use of different epitope prediction tools and further on evaluated in terms of immunogenicity. All of them revealed to be immunodominant (≥20% response rate) and four of them were classified as highly immunodominant (Table 1). Subsequent to a short-term *in vitro* stimulation, the frequencies of T cells specific to the newly identified CTL epitopes in healthy donors were lower in comparison to the known latent EBNA3A-derived A*03_EBNA3A_RLRA_. However, the long-term *in vitro* stimulation period of seven days homogenously confirmed the epitopes’ potential to expand EBV-specific T cells shown by an increase in the respective T-cell frequencies. Despite the higher frequencies ensuing short-term *in vitro* stimulation, A*03_EBNA3A_RLRA_ is less appropriate for a long-term *in vitro* stimulation of peptide-specific CTLs than the newly identified epitopes, in particular the four highly immunodominant ones. The identification of best-suited T-cell donors (e.g. HSCT, family or unrelated third-party T-cell donors) is predominantly based on the respective starting frequencies, but the T-cells’ potential to expand and to provide the most effective subsets as well as phenotypes is also essential to improve the therapeutic effect of the adoptively transferred CTLs [17, 57].

In this context, cytotoxic CD8^+^ T cells specific to the highly immunodominant epitopes revealed high proportions of both T_EM_ and T_EMRA_. T_EM_ were shown to have the highest effector function in terms of viral clearance [59, 60]. Moosmann *et al.* demonstrated that CD8^+^ T cells after being transferred either unveiled a CCR7^+^CD45RA^-^ central memory or a CCR7^-^CD45RA^+^ terminal effector phenotype and both subsets were consistent with long-term T-cell persistence and capable of controlling viral reactivations after PTLD remission [17]. Correspondingly, the immunophenotype of the cytotoxic CD8^+^ T cells specific to the four highly immunodominant epitopes unveiled to be essential to an effective adoptive immunotherapy.

### Proof of clinical relevance by monitoring EBV-specific T cells in patients suffering from EBV-associated disease

The clinically relevant natural processing and presentation of three of the highly immunodominant epitopes (A*03_BILF2_VTLA_, A*03_BcRF1_FLLA_, A*03_BALF3_QVAT_) was confirmed by peptide-specific T-cell frequencies detected in HLA-A*03:01^+^ patients with EBV-associated PTLD. Although a detailed follow up in patients was not feasible due to material shortage, it was interesting that the highly variable peptide-specific CTL frequencies were similar to those in healthy donors and will therefore need to be more thoroughly investigated in larger patient and donor cohorts. In this context, the cytolytic activity of these peptide-specific CTLs against tumor cells of HLA-A*03:01^+^ patients with EBV-associated PTLD should additionally be reaffirmed in future studies.

### Clinical relevance of the identification of a broad pathogen-specific epitope repertoire for adoptive immunotherapy

In addition to the peptides’ capacity to elicit functional T-cell-mediated immune responses against EBV, their aptitude to reinforce commercially available peptide pools was equally investigated. In line with the findings of Nowakowska *et al.* [58], the results of this study clearly demonstrated the reinforcement of the pool's stimulating efficacy when a certain mixture of the novel epitopes is added to the peptide pool EBV_Consensus. Since a higher number of epitopes in such a mixture, particularly of those restricted to one HLA-allele, might raise concerns about antigenic competition, we examined three different mixtures [61]. The CSA, a small-scale technique related to the large-scale CliniMACS system for the manufacturing of clinical-grade antigen-specific T cells, showed a step-by-step enhancement of the EBV-specific IFN-γ^+^CD3^+^ T-cell stimulating efficacy in response to EBV_Consensus+3P_MIX_ (17.2%), EBV_Consensus+4P_MIX_ (28.8%) and EBV_Consensus+11P_MIX_ (174.4%) as compared to the mere EBV_Consensus (Figure 6D). Based on this gradual enhancement, antigenic competition within the mixture is not suspected. Nonetheless, as the addition of three highly immunodominant peptides (EBV_Consensus+3P_MIX_) already achieved on average a 22.7 higher percentage value regarding the IFN-γ^+^CD8^+^ T cells in comparison to the mere EBV_Consensus prior to the enrichment (Figure 6D), this mixture represents the safest option to obviate any possible antigenic competition. IFN-γ^+^CD8^+^ T cells specific to EBV_Consensus+3P_MIX_ showed a T-cell phenotype with high proportions of T_EM_ and T_EMRA_ (78.5%) before the enrichment, hence essential for an effective EBV-clearance. Thus, the addition of a combination of the newly identified epitopes to EBV_Consensus revealed to enhance the stimulating efficacy of the mere pool without causing antigenic competition.

Apart from predominantly inducing an EBV-specific IFN-γ^+^CD8^+^ T-cell response, relevant to an immediate immune reconstitution and a functional EBV clearance [62–64], frequencies of IFN-γ^+^CD4^+^ T cells were also detected following the stimulation with the assessed mixtures, thus suggesting that the nona- or decamers might also function as efficient CD4^+^ T-cell epitopes when 2 to 4 amino acids were added to the respective sequences. As the CD4^+^ T-cell immunity is essential for long-term immunity, direct antiviral effector functions and for sustaining the CD8^+^ mediated immune response, particularly memory functions [11, 58, 65], these findings could additionally result in a further enhancement of the efficacy of the newly identified EBV-derived peptides and of the three mixtures [11, 65].

Since higher CD4^+^ T-cell frequencies in the infused EBV-specific T-cell products have furthermore been related to a better response [24], the best-suited ratio of CD8^+^ to CD4^+^ T-cells is to be identified in future studies, so that a certain, best functional ratio of both T-cell subsets can deliberately be induced to reinforce the adoptive transfer.

### Stable binding of the newly identified epitopes to pMHC complexes

In addition to proving immunogenicity, cytotoxic activity and clinical relevance of the newly identified EBV-specific T-cell epitopes, their binding efficiency and the stability of their pMHC complexes were furthermore assessed. The resultant findings of the *in vitro* Flex-T and T2 binding/dissociation assays correlated and mostly led to similar findings as the respective *in silico* predicted scores of the constantly improved epitope prediction tools [39, 42, 66]. Their algorithms enable a first selection and ranking based on predictions regarding the most pivotal aspects of a natural peptide presentation [39, 41, 42, 66, 67], but neither do they guarantee the definite accuracy of the epitope's behavior and its immunogenicity in the *in vivo* setting nor do they reflect one-to-one correlations with either *in vivo* or experimental data.

The peptide A*03_BILF2_VTLA_ for instance demonstrated a similar ability to stabilize pMHC complexes on the cell surface of T2 cells at time point t_3_ likewise to the one of the reference peptide A*03_EBNA3A_RLRA_, although the predicted half-time dissociations of both peptides varied strongly (NetMHCstab, www.cbs.dtu.dk/services/NetMHCstab-1.0). A significant consequence of this aforementioned incongruity is the fact that peptide sequences might not be stable enough to survive the individual steps of the multimer synthesis. Thus, the synthesis of peptide-specific pMHC multimers useful for the distinct detection might fail, although respective peptide-sequences have been isolated from *in vivo* and elicited an EBV-specific immune response as detected by *in vitro* T-cell immunoassays.

For instance, specific T cells against the peptide A*03_BILF2_LIIP_ have been detected in 55% of the healthy donors of this study, nevertheless its multimer synthesis failed, possibly due to the sequence's particular composition of amino acids since it consists of the highest number of seven hydrophobic amino acids that easily dissociate and not of one of the preferred residues at P9. Although it has been suggested that two preferred anchors represent a condition for a stable MHC class I interaction [68], A*03_BILF2_LIIP_ has not unveiled the fastest dissociation in the *in vitro* dissociation assay and manifested the highest peptide-binding affinity at time point t_1_ (Figure 4B).

In this context the Flex-T technology reveals to be suitable, as pMHC monomers can be generated for a peptide of interest, subsequently be multimerized and used for staining peptide-specific CD8^+^ T cells. This method allows both the assessment of pMHC complex stability for individual peptides and a fast generation of the corresponding pMHC multimer. As the generation of these multimers is immediately carried out before the T-cell staining, this technology might enable generating multimers for pMHC complexes, which are characterized by a fast dissociating stability and might therefore allow the analysis of their respective T-cell populations. These findings highlight the unavoidable disparity between the immunogenicity in the *in vivo* setting, the synthetic production and the *in silico* approach as for the peptide binding and stability.

### Future prospects: Establishment of the most suitable EBV-specific peptide pool for immunomonitoring and adoptive T-cell therapy

The experimental approach being described has proven to be a well-suited procedure to identify further HLA-restricted epitopes, consequently appropriate to enhance the stimulating efficacy of an established peptide pool as well as to broaden its HLA-coverage. This might overcome current limitations of the adoptive immunotherapy's success regarding EBV-infections, -reactivations or EBV-associated PTLD and possibly reduce the risk of tumor escape mutants as the latter might subsequently occur to the antigenic stimulants with pools covering sequences from merely one EBV protein [58]. Hence, future studies should aim at the establishment of the most suitable pool of both EBV-specific peptides derived from clinically relevant EBV proteins and of high HLA diversity, enabling the accurate immunomonitoring in patients, especially in those at high risk, as well as the selection of highly pure and functional EBV-specific T cells in sufficient numbers for adoptive transfer.

## MATERIALS AND METHODS

### Study population

All experiments were performed with residual blood samples from platelet (PLT) apheresis disposables used for routine PLT collection of regular anonymous healthy donors of the Hannover Medical School (MHH) Institute for Transfusion Medicine. Informed consent was obtained from all donors as approved by the Ethics Committee of Hannover Medical School, and trial subject data were treated as confidential information protected by medical confidentiality. The donors’ HLA-class I and II alleles were identified at four-digit resolution by means of sequence-based subtyping [28] and their EBV-serology was determined by performing confirmatory Western blot assays designated to detect anti-EBV IgG antibodies (recomLine EBV IgG, Mikrogen). Peripheral blood from either HLA-A*03:01^−^EBV^+^ or HLA-A*03:01^+^EBV^−^ healthy donors served as controls.

### Lentiviral transduction of soluble HLA-A*03:01 molecules into EBV-transformed B-LCLs

Three HLA-A*03:01^+^ EBV-transformed B-LCLs (EBV^+^B-LCLs: B-LCL_024_, B-LCL_623_, B-LCL_1335_) were cultivated in RPMI1640 (Lonza) supplemented with 10% heat-inactivated fetal calf serum (FCS, Biochrom) [29]. The EBV^+^B-LCLs were transduced with lentiviral vectors expressing sHLA-A*03:01 (sA*0301.pRRL.SFFV.mcs), as previously described [30, 31]. Secretion levels of sHLA-A*03:01 were regularly determined via quantitative w6/32-antibody ELISA [32], and compared to the background level of each of the respective untransduced EBV^+^B-LCLs.

### Purification and subsequent isolation of *in vivo* HLA-A*03:01-restricted EBV-derived peptides

Highly sHLA-A*03:01-productive clones were cultivated in two-compartment bioreactors (CELLine Classic 1000, INTEGRA-Biosciences). Supernatants were regularly harvested, pooled, and used for isolation of peptides of the sHLA-A*03:01 complexes by immunoaffinity chromatography with anti-HLA-ABC monoclonal antibody (mAb) w6/32 (Serotec) [33–35]. Pool sequencing was performed by nano-LC-ESI-MS/MS analysis and mass spectrometry was done in order to identify the peptide-sequences (TopLab) [36].

### Identification of EBV-derived candidate-epitopes by different epitope prediction tools

Mascot database queries (www.matrixscience.com, [37]) were carried out using an EBV-specific database from UniProtKB (www.uniprot.org) (Supplementary Figure 1). The resultant EBV-derived peptide-sequences were preliminarily sorted, excluding sequences with lower peptide-ion-scores than ten [38]. The remaining, coarsely filtered sequences were ranked downwards and were then screened in respect to their HLA-A*03:01-restricted binding affinity and complex stability by using the epitope prediction tools NetMHC 4.0 (www.cbs.dtu.dk/services/NetMHC, [39, 40]), NetCTL 1.2 (www.cbs.dtu.dk/services/Netctl, [41]) and NetMHCstab 1.0 (www.cbs.dtu.dk/services/NetMHCstab-1.0, [42]). Peptide-sequences were ranked in accordance to their predicted %RANK (NetMHC) and a cut-off value for sequences with affinities rating below 15%RANK was applied. The prediction results for the 20 highest scoring sequences of each of the three B-LCLs and those identified as strong [SB] or weak binders [WB] (NetMHC) were subsequently verified by SYFPEITHI (www.syfpeithi.de, [43]) and ExPASy-ProtParam-tool (www.expasy.org/protparam, [44]). To obviate alloreactivity, the sequences still remaining potentially relevant were screened with regard to homologies within the human genome using BLAST-UniProtKB (www.uniprot.org/blast). Additionally, the relevant sequences were reviewed with focus on those derived from proteins associated with EBV^+^-PTLD, -latency, -reactivation and/or with potentially malignant transformation. Ultimately, the respective sequences were verified whether to reflect the HLA-A*03:01-specific peptide supermotif [45, 46].

Peptides of the eleven highest scoring EBV-specific candidate-epitopes (Table 1) were synthesized (95% purity, EZBiolab) and used for *in vitro* studies to assess their immunogenic potential.

### Short- and long-term stimulation assays to screen for effector molecule secretion in response to EBV-derived candidate-peptide

The immunogenic potential of EBV-derived peptides was evaluated by functional immunoassays. PBMCs were isolated from blood of healthy HLA-A*03:01^+^EBV^+^ donors by density gradient centrifugation. Freshly isolated PBMCs were rested overnight (at 37°C, 5% CO_2_) at a density of 1 × 10^7^ cells/well (24-well-plate, Sarstedt) prior to short- (overnight) and long-term (7 days) *in vitro* stimulation. The known HLA-A*03:01-restricted EBV-derived peptide RLRAEAQVK (A*03_EBNA3A_RLRA_, ProImmune [17, 47]) and the peptide pool PepTivator EBV Consensus (EBV_Consensus, Miltenyi Biotec) have consistently been used as referential stimulating antigens.

On day 1, cells at a density of 5 × 10^6^ cells/well (24-well-plate, at 37°C, 5% CO_2_) were stimulated with one of the eleven EBV-specific peptides (10 μg/ml) or with EBV_Consensus+4P_MIX_ (Table 1) consisting of a combination of EBV_Consensus (1μg per peptide/ml) and the four highly immunodominant peptides (10 μg per peptide/ml). Cells were expanded in TexMACS-medium (Miltenyi Biotec) supplemented with 0.5 μl/ml interleukin (IL)-2 and 1 μl/ml IL-7 (both PeproTech) over 7 days.

### Detection of the effector molecules IFN-γ and granzyme B by EliSpot, ELISA and FluoroSpot

IFN-γ EliSpot assays allow to simultaneously detect antigen-specific T cells at a single cell level based on their IFN-γ secretion in a high number of donors and to use a variety of different stimuli. The assays were performed after short- and long-term *in vitro* stimulation, as previously described [25, 27], using precoated EliSpot plates (Lophius biosciences). Negative and positive controls were carried out by using either solitary medium or 1 μg/ml staphylococcal enterotoxins B (SEB, Sigma Aldrich). Resultant findings are indicated as number of spw, representing the number of spots in the antigen well after subtracting those of the respective negative control well. A resultant value >3 spw was defined as a positive response. Results were furthermore obtained as spw/1000 CD3^+^ T cells.

The functionality of the induced T cells was also determined by detection of the secretion levels of the effector molecules IFN-γ and granzyme B in the supernatants after long-term *in vitro* stimulation using quantitative ELISA (both eBioscience). FluoroSpot assays were furthermore performed, as they are apt to simultaneously detect the secretion of IFN-γ and granzyme B (Mabtech). PBMCs were plated at a density of either 2.5 × 10^5^ (peptide pool stimulation) or 5.0 × 10^5^ cells/well (single peptide stimulation) on precoated FluoroSpot plates (in RPMI1640/10% AB serum) and subsequent to their peptide-specific stimulation, they were incubated for a period of 45 hours. To enhance the stimulation the anti-CD28 mAb was included. Negative and positive controls were carried out by using either solitary medium or anti-CD3 mAb. Apart from EBV_Consensus+4P_MIX_, (Table 1) EBV_Consensus (2.5 × 10^5^ cells/well) was either mixed with a mixture of the three EBV-derived peptides (EBV_Consensus+3P_MIX_, Table 1), revealing the highest response rates (>60%) in first preliminary screenings, or with all eleven newly identified peptides (EBV_Consensus+11P_MIX_, Table 1). Spots identified with the filter for fluorescein isothiocyanate (FITC) represented IFN-γ producing cells and spots identified by the filter for Cyanine 3 (Cy3) detected granzyme B producing cells. Results are indicated as number of spw, equal to the number of spots in the antigen well after subtracting those of the respective negative control well.

### HLA class I peptide binding and dissociation assays

Flex-T^TM^ MHC Tetramer assays (Flex-T assay) and T2 peptide binding and dissociation assays were performed to confirm peptide binding and stability of the pHLA-A*03:01-complexes.

Flex-T^TM^ MHC Tetramer assays (BioLegend) were carried out according to manufacturers’ instructions. Briefly, each of the eleven candidate-peptides was loaded into the binding site of the HLA-A*03:01 groove by using UV light source to degrade the UV-labile peptide. The exchange process was verified by a rapid streptavidin-capture ELISA. As positive controls functioned a stable HLA class I monomer with a high-affinity, non UV-labile peptide as well as pMHC monomers for the reference A*03_EBNA3A_RLRA_ and the known HLA-A*03:01-restricted CMV_IE1-specific peptide KLGGALQAK (A*03_IE1_KLGG_, ProImmune). Unloaded HLA-A*03:01 monomers as well as HLA-A*03:01 monomers loaded with HLA-A*02:01-(NLVPMVATV, A*02_pp65_NLVP_, ProImmune) and B*07:02-restricted (TPRVTGGGAM, B*07_pp65_TPRV_, ProImmune) peptides, respectively served as negative controls.

T2 peptide binding and dissociation assays were carried out as previously described [48–51]. TAP deficient T2 cells had been transfected to express membrane-bound HLA-A*03:01 [52]. 2 × 10^6^ T2 cells/ml were plated on a 96-well-plate in 100 μl serum-free RPMI1640 and stimulated with 50 μg/ml of the respective peptide and additional 5 μg/ml beta-2 microglobulin (ß2M, Sigma) for 14–18 hours (37°C, 5% CO_2_). Peptide-unloaded cells served as negative control and the known peptide A*03_EBNA3A_RLRA_ as reference. The mAb FITC-labeled anti-HLA-ABC (w6/32, AbD Serotec) was employed to evaluate the HLA class I expression levels by flow cytometry (FACSCanto 10c, FACSDiva V8.0.1 software, BD Biosciences). For pMHC-complex dissociation assays, peptide-loaded cells were washed with Phosphate-buffered saline (PBS (Lonza) after incubation and resuspended in RPMI1640/2% FCS. Aliquots of cells were collected at the indicated time points (t_1_ = 0 min, t_2_ = 60 min, t_3_ = 120 min) and analyzed by flow cytometry using anti-w6/32-FITC mAb [48]. The resultant fluorescence index (FI) was calculated as the mean fluorescence intensity (MFI) of HLA-A*03:01 on peptide-loaded and -unloaded T2 cells, respectively in order to visualize the dissociation degree at different time points. In terms of a comparative reference the unloaded T2 cells were standardized to 0.

### Enrichment efficiency of EBV peptide-specific T cells by cytokine secretion assay

The non-GMP IFN-γ CSA (Miltenyi Biotec), is a small-scale procedure on the basis of the clinical-scale CliniMACS system. Results obtained by CSA show the tendency to mirror the enrichment efficacy of clinical -grade antigen-specific T cells. CSA was performed according to the manufacturer's instructions and used (1) to verify the immunogenicity of the newly identified epitopes and (2) to predict the T-cell enrichment efficiency [53, 54]. Unstimulated cells served as negative control while A*03_EBNA3A_RLRA_ and the PepTivator EBV Consensus, respectively were used as references. PBMCs were either stimulated with the highly immunodominant candidate-peptides (A*03_BILF2_VTLA_, A*03_BcRF1_FLLA_, A*03_BALF3_QVAT_ and A*03_BILF2_LIIP_, Table 1) alone or with one of the three different mixtures of the *in vivo* isolated peptides and EBV_Consensus (EBV_Consensus+3P_MIX_, EBV_Consensus+4P_MIX_, EBV_Consensus+11P_MIX_, Table 1). Aliquots of the respective cell fractions before (origin) and after enrichment (elution) were analyzed by multicolor flow cytometry. To distinguish between naïve (T_N_: CD62L^+^CD45RA^+^), central memory (T_CM_: CD62L^+^CD45RA^−^), effector memory (T_EM_: CD62L^−^CD45RA^−^) and terminally differentiated effector memory T cells (T_EMRA_: CD62L^−^CD45RA^+^), cells were stained in addition to anti-IFN-γ-phycoerythrin mAb (PE, Miltenyi Biotec) with the following antibodies: FITC-labeled anti-CD3 mAb, allophycocyanin (APC)-labeled anti-CD8 mAb, APC-Cy7-labeled anti-CD45 mAb (all BioLegend) and Alexa Fluor (AF) 700-labeled anti-CD4 mAb, Brilliant Violet (BV) 421-labeled anti-CD62L mAb, BV 510-labeled anti-CD45RA mAb and BV 605-labeled anti-CD27 mAb (all BD Bioscience). Distribution of viable and dead cells was done by 7-aminoactinomycin (AAD)-staining. In the viable CD45^+^7AAD^−^-leukocyte-gate at least 10,000 events were acquired.

### Visualization of EBV-specific T cells by pMHC mutimer staining

The pMHC multimer staining technology allows the direct visualization of antigen-specific T cells in the blood of potential donors and patients. Staining was meant to confirm the clinical relevance of the highly immunodominant candidate-epitopes (Table 1) by detecting CD8^+^ CTL frequencies specific to the newly identified epitopes. Blood samples from HLA-A*03:01^+^ patients (*n* = 5) with EBV-associated PTLD were obtained at two different time points during their therapy: prior to receive the second, third, fourth or fifth treatment with Rituximab, after their sixth treatment with Rituximab, in follow-up care ensuing cytotoxic chemotherapy or in follow-up care subsequent to no other treatment than Rituximab. Specific CD8^+^ CTL frequencies were additionally visualized in freshly isolated PBMCs of healthy HLA-A*03:01^+^ donors (*n* = 5). To safeguard specific detection, pMHC multimer staining was also performed with cells from HLA-A*03:01^-^ donors. Multimers (PE-conjugated dextramers, IMMUDEX) for three of the four highly immunodominant EBV-derived CTL epitopes were successfully synthesized. The synthesis of A*03_BILF2_LIIP_ unfortunately failed. The general nonsense dextramer was applied to exclude false-positive results, while the A*03_EBNA3A_RLRA_ dextramer served as reference. Phenotypic T-cell characterization was carried out using the following mAbs: anti-CD3-FITC, anti-CD8-APC, anti-CD45-APC-Cy7 (all BioLegend), anti-CD4-AF700, anti-CD62L-BV421, anti-CD45RA-BV510 and anti-CD27-BV605 (all BD Bioscience). For viability staining 7-AAD was included. Gating was carried out in accordance with the light scatter properties of lymphocytes and CD3^+^ T-cell populations. With at least 100,000 events in the lymphocyte gate, the focus of the gating strategy was set on the CD3^+^CD8^+^ T cells in order to assess multimer^+^ T cells. Additionally, it was verified whether a well-defined cell population could be identified. Resultant frequencies are given as percent values after subtracting those of the respective general nonsense dextramer.

### Statistical analysis

Statistical analysis was performed by Prism v5.02 software (GraphPad). Generated data were analyzed using either non-parametric Mann-Whitney *U*-tests or one-way ANOVA and Bonferroni tests. Results were presented as means ± standard deviation (SD) and levels of significance expressed as *p*-values (^*^p < 0.05, ^**^p < 0.01, ^***^p < 0.001). Significant differences have been detected in comparison to the referential antigens (A*03_EBNA3A_RLRA_, EBV_Consensus).

## SUPPLEMENTARY MATERIALS FIGURES


