# Supplementary material for: Personalized adoptive immunotherapy for patients with EBV-associated tumors and complications: Evaluation of novel naturally processed and presented EBV-derived T-cell epitopes
Source: Oncotarget. 2017 Dec 21;9(4):4737–57. doi: 10.18632/oncotarget.23531 (PMC5797009; doi:10.18632/oncotarget.23531)
Supplement: Supplementary file 1 [file oncotarget-09-4737-s001.pdf]

Personalized adoptive immunotherapy for patients with EBV-associated tumors and complications: Evaluation of novel naturally processed and presented EBV-derived T-cell epitopes

SUPPLEMENTARY MATERIALS

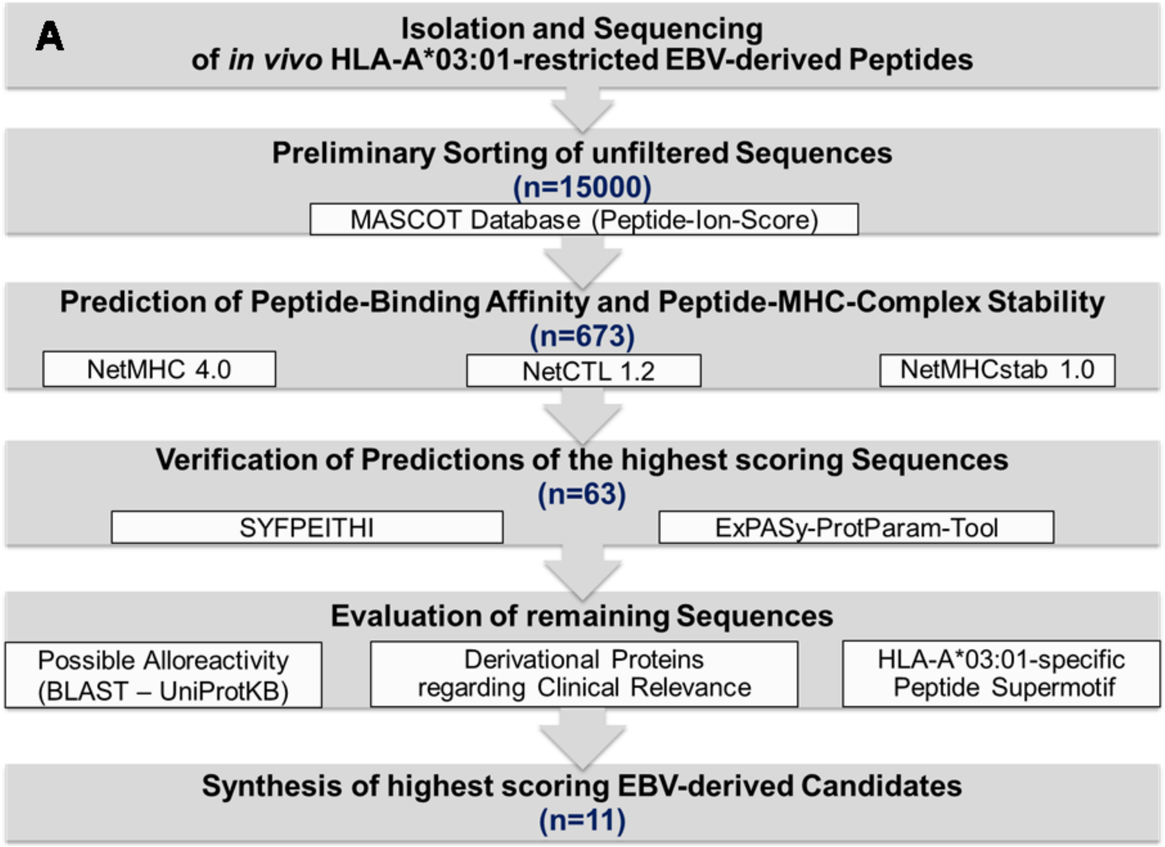

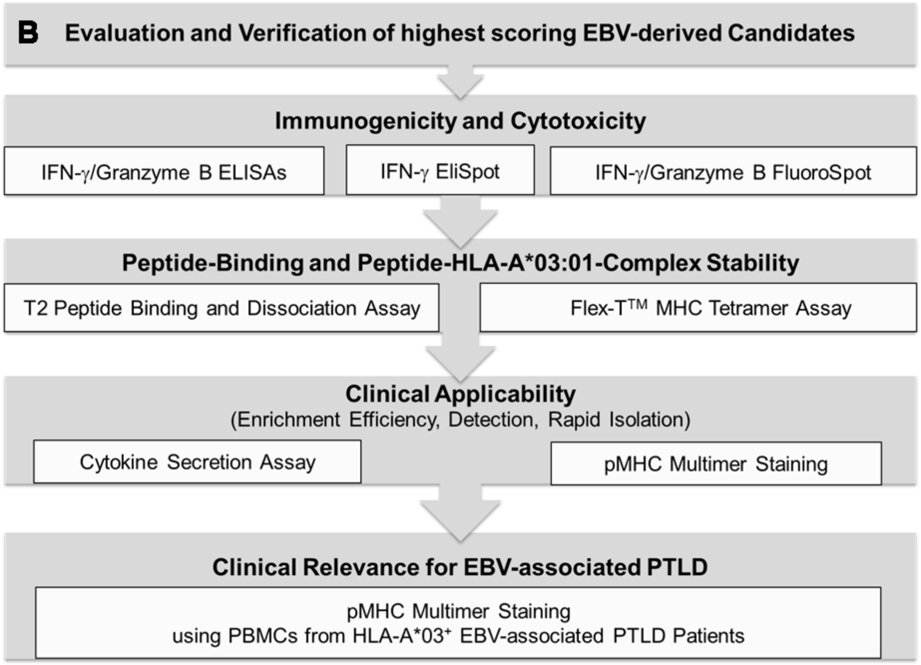

Supplementary Figure 1: (**A**–**B**) Overview of the experimental approach for (A) the identification and (B) the evaluation of potentially relevant EBV-specific T-cell epitopes. The experimental approach of this study was divided into two main parts. (A) EBV-derived peptide-sequences *in vivo* isolated from sHLA-A*03:01-transduced B-LCLs. Their potential as relevant EBV-specific T-cell epitopes was assessed by a combination of different epitope prediction tools. Homologies within the human genome were obviated (BLAST-UniProtKB), sequences were reviewed in respect to the HLA-A*03:01-supermotif and derivational proteins were accounted. (B) Sequences of the EBV-candidates ranked highest in part (A) were synthesized as peptides. Immunogenicity and clinical relevance of the newly identified EBV-derived T-cell epitopes were evaluated by established T-cell immunoassays.

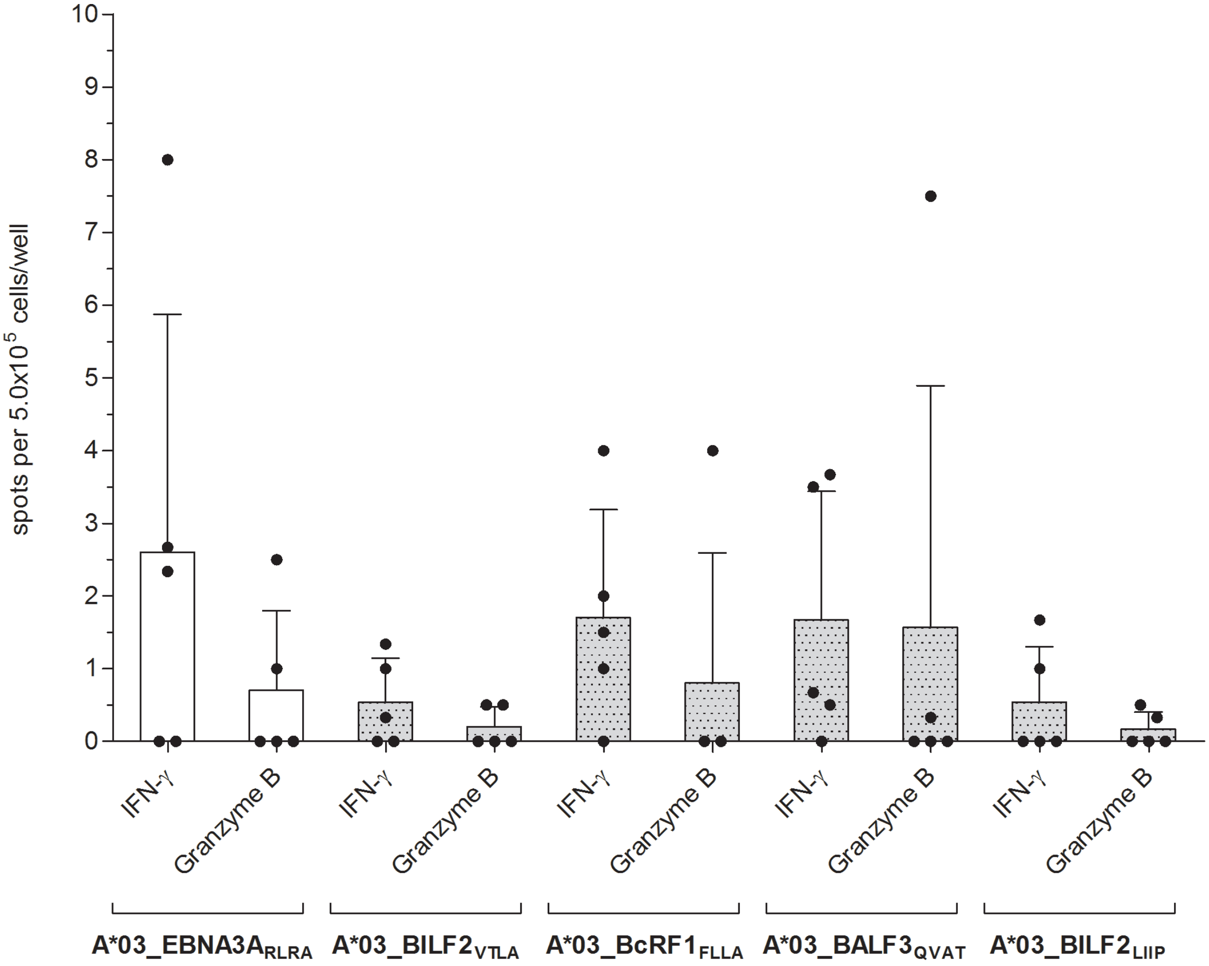

Supplementary Figure 2: Evaluation of the immunogenic and cytotoxic activity of the peptide-induced EBV-specific T **cells.** The cytotoxic activity of the peptide-induced EBV-specific T cells was investigated by means of IFN-γ and granzyme B FluoroSpot assays. PBMCs from healthy HLA-A*03:01-positive, EBV-seropositive donors (*n* = 5) were stimulated with one of the four highly immunodominant peptides (Table 1). The known HLA-A*03:01-restricted peptide A*03_EBNA3A$_{RLRA}$ served as referential antigen. Spots identified with the filter for FITC represented IFN-γ producing cells and spots identified by the filter for Cy3 detected granzyme B producing cells. Results are given as the number of spots per well (spw), representing the number of spots in the antigen well after subtracting those of the respective negative control well. Results are displayed as individual results and as means and standard deviation (SD).

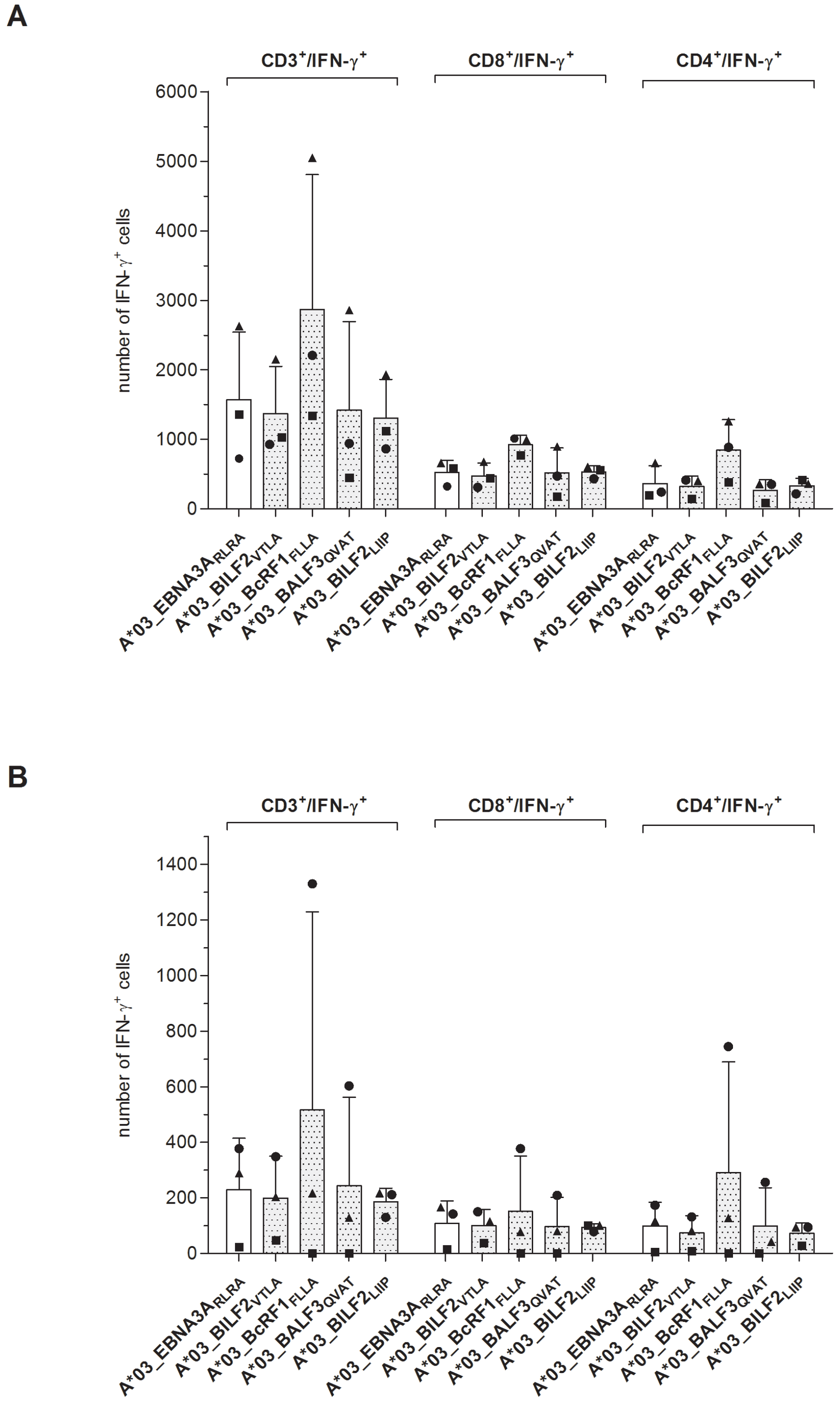

Supplementary Figure 3: (**A–B**) Evaluation of the eligibility of the highly immunodominant HLA-A*03-restricted EBV-derived peptides for clinical application – by means of CSA. PBMCs from healthy donors (n = 3) were stimulated with one of the four highly immunodominant EBV-peptides. Respective frequencies of the IFN-γ-secreting cell fractions before (origin) and after the enrichment (eluate) were determined by multicolor flow cytometry. Frequencies in the 'origin' evaluated in comparison to (A) the number of the IFN-γ-secreting T cells in response to the EBV-derived reference peptide A*03_EBNA3A$_{RLRA}$. (B) The efficiency of the IFN-γ-specific enrichment via the magnetic labeling of IFN-γ-secreting cells is separately shown for each of the assessed peptides by the respective number of the IFN-γ-secreting T cells. Results are displayed as individual results and as means ± SD.

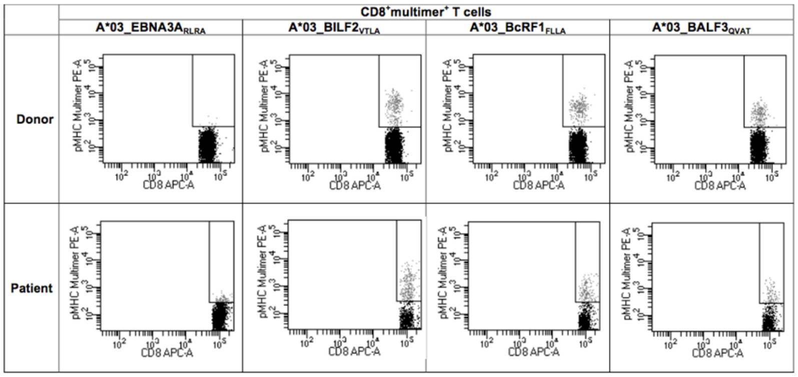

Supplementary Figure 4: (**A–D**) Exemplaric dot plots of peptide-specific CD8$^+$multimer$^+$ T-cell frequencies in one healthy donor and one EBV$^+$PTLD-patient. The frequencies of CD8$^+$multimer$^+$ T cells specific to one of the highly immunodominant EBV-derived peptides (A*03_BILF2$_{VTLA}$, A*03_BcRF1$_{FLLA}$, A*03_BALF3$_{QVAT}$) and to the known immunodominant EBV-derived peptide (A*03_EBNA3A$_{RLRA}$) were visualized by pMHC multimer staining in both PBMCs from healthy donors and patients with EBV-associated PTLD. The subsequent analysis was carried out by multicolor flow cytometry. Exemplaric dot plots regarding the peptide-specific CD8$^+$multimer$^+$ T cells of one healthy donor and of one patient are individually illustrated for each of the peptides.
